# Population Pharmacodynamic Modeling Using the Sigmoid E_max_ Model: Influence of Inter-individual Variability on the Steepness of the Concentration–Effect Relationship. a Simulation Study

**DOI:** 10.1208/s12248-020-00549-7

**Published:** 2020-12-24

**Authors:** Johannes H. Proost, Douglas J. Eleveld, Michel M. R. F. Struys

**Affiliations:** 1 Department of Anesthesiology, University Medical Center Groningen, University of Groningen, Hanzeplein 1, 9713 GZ Groningen, the Netherlands; 2 Department of Pharmacokinetics, Toxicology and Targeting, Groningen Research Institute of Pharmacy, University of Groningen, Groningen, the Netherlands; 3 Department of Anesthesia, Ghent University, Gent, Belgium

**Keywords:** pharmacokinetic-pharmacodynamic modeling, sigmoid Emax model, inter-individual variability, simulation

## Abstract

The relationship between the concentration of a drug and its pharmacological effect is often described by empirical mathematical models. We investigated the relationship between the steepness of the concentration–effect relationship and inter-individual variability (IIV) of the parameters of the sigmoid E_max_ model, using the similarity between the sigmoid E_max_ model and the cumulative log-normal distribution. In addition, it is investigated whether IIV in the model parameters can be estimated accurately by population modeling. Multiple data sets, consisting of 40 individuals with 4 binary observations in each individual, were simulated with varying values for the model parameters and their IIV. The data sets were analyzed using Excel Solver and NONMEM. An empirical equation (Eq. (11)) was derived describing the steepness of the population-predicted concentration–effect profile (γ*) as a function of γ and IIV in C50 and γ, and was validated for both binary and continuous data. The tested study design is not suited to estimate the IIV in C50 and γ with reasonable precision. Using a naive pooling procedure, the population estimates γ* are significantly lower than the value of γ used for simulation. The steepness of the population-predicted concentration–effect relationship (γ*) is less than that of the individuals (γ). Using γ*, the population-predicted drug effect represents the drug effect, for binary data the probability of drug effect, at a given concentration for an arbitrary individual.

**Supplementary Information:**

The online version contains supplementary material available at 10.1208/s12248-020-00549-7.

## INTRODUCTION

The relationship between the concentration of a drug and its pharmacological effect is often described by empirical mathematical models, as a convenient method to explore this relationship quantitatively, *e.g.*, to predict the time course of drug effect by pharmacokinetic-pharmacodynamic modeling (1–3). This approach can be used for both continuous and binary (also denoted quantal or dichotomous) drug effects, and can be extended to combinations of drugs using response surface modeling to investigate the interaction of two or more drugs (4–7).

The concentration–effect relationship is usually described by the sigmoid E_max_ model. This model has a limited physiological and mechanistic basis, since it reflects the relationship between drug concentration and effect in the case that the drug effect is proportional to the receptor occupancy; in this case, the concentration at which the drug effect is 50% of the maximal effect (C50) equals the dissociation constant *K*_d_ and the slope of the concentration–effect relationship (γ) equals 1 (see “METHODS” for details). In other cases, the sigmoid E_max_ model should be considered as an empirical equation that describes the concentration–effect relationship sufficiently accurately, as has been shown in numerous papers over the last four decades.

An interesting characteristic of the sigmoid E_max_ model is that the concentration–effect relationship is close to that of the cumulative log-normal distribution (8). This implies that it can be used in cases where the concentration–effect is likely to follow a cumulative log-normal distribution, *e.g.*, in the case of binary responses, where the probability of response is modeled as a function of drug concentration. However, the similarity of the sigmoid E_max_ model and the cumulative log-normal distribution has not been investigated in detail in pharmacological literature.

The relationship between the concentration of anesthetic drugs and binary measures of depth of anesthesia, *e.g.*, response to a standardized stimulus, is often rather steep. However, the reported steepness varies widely between studies, even for the same stimulus and using a similar study design (9–12). In some studies, the population analysis resulted in a final analysis including inter-individual variability (IIV) in C50, based on the lower value for the objective function value (10, 11), but in another study no IIV in C50 could be identified (9). This results in remarkable differences in γ, which was reported as 3.46 for propofol (without IIV), 17.6 for sevoflurane + propofol (with 31–32% IIV), and 7.41 for sevoflurane (with 20% IIV). A simultaneous analysis of the data of these three studies revealed that the steepness of the concentration–effect relationship is, among other factors, dependent on the inclusion or exclusion of IIV in model parameters (12). Exclusion of IIV in C50 resulted in a lower value for steepness.

This phenomenon has been investigated in two papers (3, 13), demonstrating that, when data from multiple patients is naively pooled, the estimates of γ may be biased, and the 95% confidence intervals may not contain the true value. The authors stated: “We believe that estimates of γ from studies in which data from multiple patients was naively pooled must be viewed with suspicion. In this type of analysis, intra-patient variability (embodied in the parameter γ) cannot be distinguished from inter-patient variability. Accurate estimates of γ necessitate methods of analysis that take inter-patient variability into account.” However, they did not provide insight in how such a study design should be chosen.

It is the aim of this paper (procedure 1) to describe quantitatively the relationship between the steepness of the concentration–effect relationship and IIV in the model parameters, and (procedure 2) to investigate whether IIV in the model parameters can be estimated by population analysis with data obtained from study designs as used in reported clinical research studies. To this purpose, several simulation studies were performed. Finally, we discuss the question whether or not IIV should be included in population modeling of binary responses.

## METHODS

We describe the procedures for a continuous drug effect as well as for a binary drug effect. For convenience, we consider here the situation where only one drug is administered. Once the principle has been developed, the method can be extended for any combination of drugs, including combinations of hypnotic and opioid drugs (12).

### Sigmoid E_max_ Model

If we assume that the drug effect in an individual can be predicted from the sigmoid E_max_ model, the drug effect P is defined:

1 $$ P\kern0.5em =\kern0.5em \frac{C^{\gamma }}{C{50}^{\gamma}\kern0.5em +\kern0.5em {C}^{\gamma }} $$

where C is the effect-site concentration, C50 is the effect-site concentration resulting in P = 0.5, and γ is a dimensionless model parameter, reflecting the steepness of the concentration–effect relationship. In the case of a binary drug effect, P reflects the probability of a drug effect at drug concentration C.

**Fig. 1:**
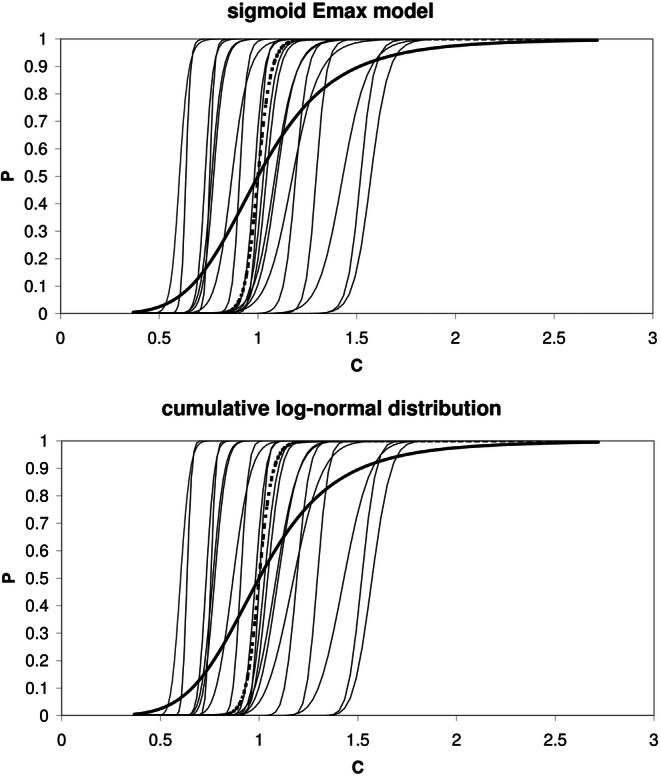
Concentration–*P* relationship of 20 simulated individuals (thin lines) using the sigmoid E_max_ model (upper panel) and cumulative log-normal distribution (lower panel. Typical individual (dashed line) and population prediction (solid line). C50 = 1 (arbitrary unit); γ = 30 (sigmoid E_max_ model); σ = 0.0567 (cumulative log-normal distribution); ω_C50_ = 0.1; ω_γ_ = 0.1

Throughout this paper, it is assumed that the model parameters are log-normally distributed within the population. An example is shown in Fig. 1 (upper panel, thin lines). As a result of IIV in C50 and γ, the concentration–effect relationship is different for each individual.

### Cumulative Log-Normal Distribution

If we assume that P in an individual can be predicted from the cumulative log-normal distribution function, P is defined:

2 $$ P\kern0.5em =\kern0.5em \Phi (z) $$

where Φ(z) is the cumulative normal distribution function (ranging from 0 for z = − ∞ to 1 for z = + ∞), and z is the normalized distance to the mean; for a log-normal distribution:

3 $$ z\kern0.5em =\kern0.5em \frac{\ln (C)\kern0.5em -\kern0.5em \ln (C50)}{\sigma } $$

where σ is the (dimensionless) standard deviation of the log-normal distribution, which determines the steepness of the concentration–effect relationship.

**Fig. 2:**
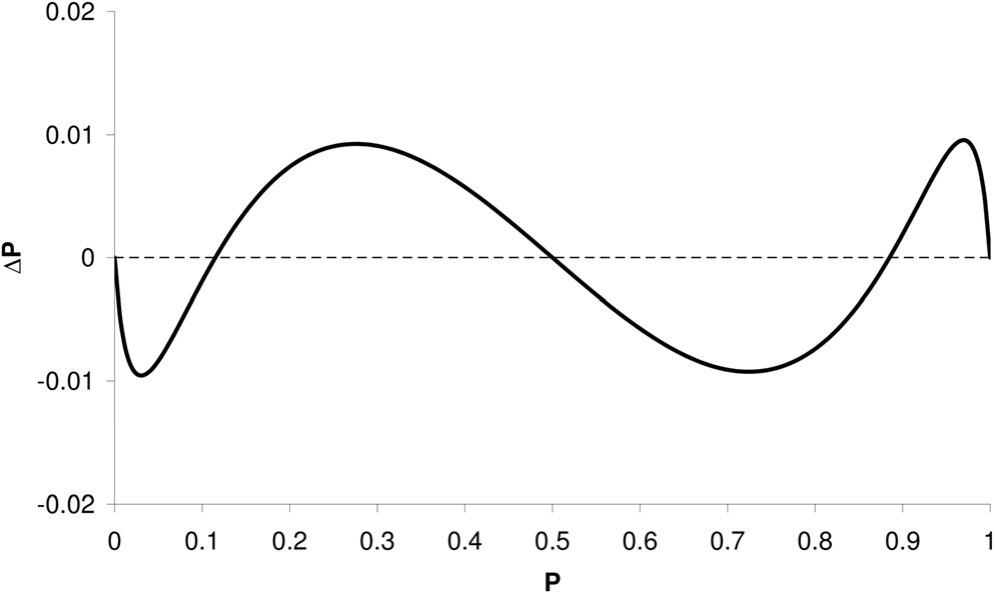
Difference (Δ*P*) between the profiles of *P* obtained with sigmoid E_max_ model and *P* obtained with cumulative log-normal distribution, using γ* = 5.27 calculated using Eq. (11) and σ* = 0.323 using Eq. (10), respectively. Parameter values as in Fig. 1: C50 = 1 (arbitrary unit); γ = 30 (sigmoid E_max_ model); σ = 0.0567 (cumulative log-normal distribution); ω_C50_ = 0.1; ω_γ_ = 0.1

An example is shown in Fig. 1 (lower panel, thin lines). As a result of IIV in C50 and σ, the concentration–effect relationship is different for each individual. Note the similarity between the profiles in both panels of Fig. 1; Fig. 2 shows that the difference in P (ΔP) is less than 0.01 (1%) over the entire scale and is minimal for P = 0.5 and at P = 0.115 and P = 0.885.

### Comparison of Sigmoid E_max_ Model and Cumulative Log-Normal Distribution

A logistic approximation of the cumulative normal distribution has been described by Bowling and colleagues (8):

4 $$ P\kern0.5em =\kern0.5em \frac{1}{1\kern0.5em +\kern0.5em \exp \left(-a\cdot z\right)} $$

where z is a normally distributed random variable with mean zero and variance one, and a is a constant. The best agreement between the cumulative log-normal distribution (Eqs. (2) and (3)) and the logistic function (Eq. (4)) was found for a = 1.702 (8). For our purposes, a constant value of 1.7 was found to be sufficiently accurate (and more convenient in practice) and was used throughout this article.

Equating P from Eqs. (1) and (4), it follows that

5 $$ {\left(\frac{C}{C50}\right)}^{\gamma}\kern0.5em =\kern0.5em \exp \left(a\cdot z\right) $$

Combining Eqs. (1), (3), and (5), it follows for a = 1.7

6 $$ \gamma =\kern0.5em \frac{1.7}{\sigma } $$

Using Eq. (6), σ (describing the steepness of the concentration–effect relationship in the cumulative log-normal distribution) can be converted to γ (describing the steepness in the sigmoid E_max_ model), and vice versa. Note that Eq. (6) is an approximation, since both distributions are close to each other, but not identical. However, as shown in Fig. 2, the difference between the two distributions is small and likely not distinguishable when applied to clinical data.

### Influence of IIV in Population Analysis

The influence of IIV in population analysis is shown in Fig. 1. Each individual (thin lines) shows a steep concentration–effect relationship, with a different steepness due to IIV in γ (upper panel) or σ (lower panel), whereas IIV in C50 results in a shift along the concentration axis. The steepness of the concentration–effect relationship of the typical individual (dashed line) is similar to that of the individuals. However, the population-predicted concentration–effect relationship, which can be calculated by averaging P at each concentration value (solid line), is less steep than that of the individual patients. This population-predicted P represents the probability of a drug effect at a given concentration averaged across all individuals, which may be interpreted as the probability of a drug effect at a given concentration for an arbitrary individual. The steepness of the population-predicted P may be expressed by the sigmoid E_max_ model (using symbol γ*, to discriminate from the model parameter γ) or cumulative log-normal distribution (using symbol σ*, to discriminate from the model parameter σ) and is a function of γ (or σ) and the IIV in C50 and γ (or σ).

### Procedure 1: Population Predictions for σ* and γ*

Using the similarity between the probability of drug effect according to the sigmoid E_max_ model and the cumulative log-normal distribution, an equation for the relationship between γ and IIV in C50 and γ on the population-predicted γ* was derived in a Monte Carlo simulation study.

The combined effect of γ and IIV in C50 and γ on the population-predicted γ* cannot be derived mathematically. However, the combined effect of σ and IIV in C50 and γ on σ* may be evaluated from the principle that variances sum up. According to this principle, we postulated that the variance σ*^2^ may be approximated by the following expression, summing σ^2^ and the variances ω_C50_ and ω_γ_ (using the symbol omega in NONMEM for inter-individual variance) and the interaction of ω_C50_ and ω_γ_:

7 $$ \sigma {\ast}^2=\kern1em {p}_1\kern0.5em \cdot \kern0.5em \frac{\sigma^2}{\gamma^{\mathrm{p}5}}\kern1em +\kern1em {p}_2\kern0.5em \cdot \kern0.5em \frac{\omega_{\mathrm{C}50}}{\gamma^{\mathrm{p}6}}+\kern1em {p}_3\kern0.5em \cdot \kern0.5em \frac{\omega \gamma}{\gamma^{\mathrm{p}7}}\kern1em +\kern1em {p}_4\kern0.5em \cdot \kern0.5em \frac{\omega_{\mathrm{C}50}\kern0.5em \cdot \kern0.5em \omega \gamma}{\gamma^{\mathrm{p}8}} $$

where σ is defined by Eq. (6) and p_i_ (i = 1 to 8) are constants, which were estimated using the numerical procedure described below. Note that all parameters and constants in Eq. (7) are dimensionless.

The population-predicted steepness of the concentration–effect relationship (γ*) can be obtained from the analogue of Eq. (6)

8 $$ \gamma \ast =\kern0.5em \frac{1.7}{\sigma \ast } $$

The population-predicted steepness γ* from Eq. (8) can be used to calculate the profile of population-predicted P.

The value of γ* calculated from Eq. (8) is lower than that of the typical individual (Fig. 1) since it takes into account the population variability of C50 and γ.

#### Estimation of Constants p_i_ in Eq. (7) by Monte Carlo Simulation

The distributions as shown in Fig. 1 cannot be used directly for population predictions. For population predictions, we need to derive the relationship between the drug concentration and P within a (large) population of individuals. This was done by estimating the constants p_i_ in Eq. (7) with the following Monte Carlo simulation procedure:

1. A large number of individuals (here 10,000) was simulated, with randomly drawn values for C50 and γ using the sigmoid E_max_ model. Separate sets were generated for each combination of population values for C50 (arbitrary unit with value 1 for each set), γ (values 0.5, 1, 2, 3, 5, 10, 20, 30, 50), ω_C50_ (0, 0.05, 0.1, 0.2, 0.3, 0.5) and ω_γ_ (0, 0.05, 0.1, 0.2, 0.3, 0.5); the total number of generated sets was 1 × 9 × 6 × 6 = 324.
2. For each simulated individual, P was calculated over a wide range of concentrations, using Eq. (1) (sigmoid E_max_ model) or Eq. (2) (cumulative log-normal distribution). The concentrations were equally spaced on a logarithmic scale, according to the following equation:

9 $$ {C}_i\kern1em =\kern1em C50\kern0.5em \cdot \kern0.5em \exp \left(\frac{i\kern0.5em \cdot \kern0.5em \ln \left(1\kern0.5em /\kern0.5em p\kern0.5em -\kern0.5em 1\right)}{n\kern0.5em \cdot \kern0.5em \gamma \ast}\right) $$

where C_i_ is the *i*th concentration (i = − 50 to 50), C50 is the typical C50 value (population mean), p is the lower range of probability levels tested (here p = 0.01, implying that P for the lowest and highest concentration is 0.01 and 0.99, respectively, and γ* is defined by Eqs. (7) and (8).

3. When simulating binary data, the actual binary status of each patient for each calculated P was simulated by drawing a random number between 0 and 1: if P was above this value, the drug effect was considered to be present (assigned a value of 1); if P was below this value, the drug effect was considered to be absent (assigned a value of 0). However, it was confirmed by simulations that this dichotomy step may be left out, since it results in the same values for P over the entire concentration range, with much better precision, avoiding loss of information in the dichotomy step. Therefore this step was skipped in all presented results.
4. At each concentration point, the population-predicted P was calculated as the sum of P for each individual (obtained by Monte Carlo simulation in step 3), divided by the number of individuals.
5. At each concentration point, the population-predicted P was also calculated using Eq. (1) with the population-predicted γ* calculated using Eqs. (7) and (8).
6. The best-fitting values of C50 and γ* were obtained by minimizing the sum of the squared differences between P calculated in steps 4 and 5 over the entire concentration range (Eq. 9), using the Solver in Excel 2019 (Microsoft, Redmond, Washington USA).
7. From the 324 combinations of γ, ω_C50_ and ω_γ_ (step 1), the best fitting values of p_i_ (i = 1 to 8) in Eq. (7) were obtained by minimizing the sum of the squared differences between the logarithms of γ* obtained in step 4 and γ* calculated from Eqs. (7) and (8), using the Solver.

To calculate the concentration values used in the simulations from Eq. (9), γ* must be known, so the constants p_i_ in Eq. (8) must be known, whereas these simulations are intended to obtain the empirical values of p_i_. To solve this chicken-and-egg problem, initial values of the constants p_i_ were obtained by a stepwise procedure: (1) p_1_ and p_5_ were solved from the simulation sets with varying values of γ, and fixed values ω_C50_ = ω_γ_ = 0 and fixed values p_2_ = p_3_ = p_4_ = p_6_ = p_7_ = p_8_ = 0; (2) p_2_ and p_6_ were solved from the simulation sets with varying values of γ and ω_C50_, and fixed value ω_γ_ = 0 and fixed value p_3_ = p_4_ = p_7_ = p_8_ = 0; (3) p_3_ and p_7_ were solved from the simulation sets with varying values of γ and ω_γ_, and fixed value ω_C50_ = 0 and fixed value p_4_ = p_8_ = 0; (4) p_4_ and p_8_ were solved from the simulation sets with varying values of γ, ω_C50_ and ω_γ_; (5) Finally, all constants p_i_ were estimated simultaneously from all data, until the estimated values of p_i_ were similar to that used in the preceding step.

### Procedure 2: Simulation and population analysis using NONMEM

To validate the relationship between γ, ω_C50_, ω_γ_ and γ* in a situation comparable to that in reported clinical studies (9–12), a series of simulations were performed with the sigmoid E_max_ model, followed by population analysis using NONMEM version 7.3.0 (Icon Development Solutions, Hanover, MD).

Synthetic data sets (1000 repetitions for each combination of model parameters and IIV) were generated, consisting of 40 individuals each, drawn from the model parameters with or without IIV in C50 and γ. The population parameters were varied: C50 (arbitrary value 1 for each set), γ (1, 5, 30), ω_C50_ (0, 0.02, 0.05, 0.1, 0.2, 0.5) and ω_γ_ (0, 0.02, 0.05, 0.1, 0.2, 0.5; excluding combinations with ω_γ_ unequal to 0 and ω_C50_, to avoid excessive computational burden), resulting in 48 data sets.

Four drug levels in each individual were chosen for each combination of model parameters in such a way, that the population-predicted values of P, calculated from Eq. (1) using the “true” population C50 and γ* from Eq. (10) were 0.10, 0.25, 0.75, and 0.90, respectively. These four values were chosen to cover a large part of the informative part of the concentration–effect profile and to guarantee that the information density was similar in each set of simulations.

At each of the four drug levels, P was calculated from the individual C50 and γ and drug level. A random number between 0 and 1 was generated; if P was above this value, the drug effect was considered to be present (assigned a value of 1); if not, the drug effect was considered absent (assigned a value of 0).

The simulated data sets were analyzed using the sigmoid E_max_ model three times, with IIV in C50 and γ, IIV in C50 only, and without IIV, respectively. The resulting population values of C50 and γ* were evaluated by comparing the median value of 1000 replications with the “true” value of C50 and the value of γ* calculated using Eq. (11).

### Calculations

The simulations and population analyses were performed using NONMEM version 7.3.0. The following code was used to control the estimation step: $ESTIMATION SIG=4 MAX=9999 METHOD=COND LAPLACE LIKELIHOOD. For the covariance matrix, the default setting was used. Other calculations were performed in Excel 2019 (Microsoft, Redmond, Washington USA).

## RESULTS

### Procedure 1: Population Predictions for σ* and γ*

To estimate the constants p_i_ in Eq. (7), a series of simulations were performed using the Monte Carlo procedure described in the methods section. For each combination of γ, ω_C50_, and ω_γ_, the population predictions for C50 and γ were estimated by fitting Eq. (7) to the average of 10,000 simulated probability profiles. The estimated γ* was compared to γ* obtained from Eq. (7). A selection of results is presented in Table I (complete results in supplemental table S1). From these results, the best fitting values, rounded to practical values, were p_1_ = 1, p_2_ = 1, p_3_ = 1.25, p_4_ = 2.5, p_5_ = 0, p_6_ = 0, p_7_ = 2, p_8_ = 1, resulting in the following equation:

**Table I:** Comparison of γ and σ estimated by fitting to the sum of 10,000 simulated probability profiles of the sigmoid E_max_ model (γ_mc_) and cumulative log-normal distribution (σ_mc_) and calculated from Eqs. (11) (γ*) and (10) (σ*), respectively (complete results in supplemental table S1)

Simulation			Estimation	Calculated from Eq. (11)		Estimation cumulative log-normal distribution	Calculated from Eq. (10)	
Sigmoid E_max_ model			Sigmoid E_max_ model					
γ	ω_C50_	ω_γ_	γ_mc_	γ*	%diff^a^	σ_mc_	σ*	%diff^*a*^
1	0	0	1.000	1.000	0.0	1.698	1.700	0.1
1	0	0.1	0.977	0.979	0.2	1.737	1.736	−0.1
1	0.1	0	0.981	0.983	0.2	1.730	1.729	−0.1
1	0.1	0.1	0.953	0.959	0.7	1.782	1.772	−0.6
5	0	0	5.00	5.00	0.0	0.340	0.340	0.1
5	0	0.1	4.91	4.90	−0.2	0.346	0.347	0.3
5	0.1	0	3.61	3.66	1.4	0.470	0.464	−1.3
5	0.1	0.1	3.49	3.58	2.5	0.486	0.475	−2.3
30	0	0	30.0	30.0	0.0	0.0566	0.0567	0.1
30	0	0.1	29.5	29.4	−0.5	0.0575	0.0579	0.6
30	0.1	0	5.29	5.29	0.0	0.321	0.321	0.1
30	0.1	0.1	5.24	5.27	0.4	0.324	0.323	−0.3

^*a*^% difference between γ* and γ_mc_ or between σ* and σ_mc_

10 $$ \sigma {\ast}^2=\kern1.5em {\sigma}^2\kern1em +\kern1em {\omega}_{\mathrm{C}50}\kern0.5em +\kern1em 1.25\kern0.5em \cdot \kern0.5em \frac{\omega \gamma}{\gamma^2}\kern1em +\kern1em 2.5\kern0.5em \cdot \kern0.5em \frac{\omega_{\mathrm{C}50}\kern0.5em \cdot \kern0.5em \omega \gamma}{\gamma } $$

Combining Eqs. (6), (8), and (10), results in the following approximation for γ*:

11 $$ \gamma \ast =\kern1.5em \frac{1.7}{\sqrt{{\left(\frac{1.7}{\gamma}\right)}^2\kern1em +\kern1em {\omega}_{\mathrm{C}50}\kern0.5em +\kern1em 1.25\kern0.5em \cdot \kern0.5em \frac{\omega \gamma}{\gamma^2}\kern1em +\kern1em 2.5\kern0.5em \cdot \kern0.5em \frac{\omega_{\mathrm{C}50}\kern0.5em \cdot \kern0.5em \omega \gamma}{\gamma }}} $$

Note that σ* = σ and γ* = γ in the absence of IIV in C50 and γ (ω_C50_ = 0 and ω_γ_ = 0). Using Eq. (11), γ* could be predicted with a mean precision of 1.2% (root mean squared error; range − 4.4 to + 5.2%) for any of the tested combinations of γ, ω_C50_ and ω_γ_.

**Fig. 3:**
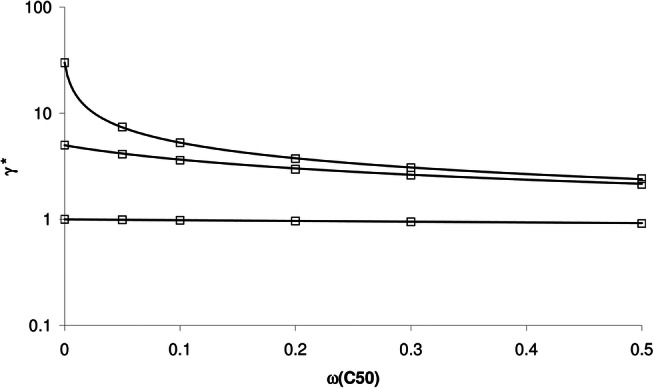
Relationship between γ* and ω_C50_ obtained by Monte Carlo simulation (open symbols), and the model prediction using Eq. (11) (solid lines), for γ = 1 (lower line and symbols), 5 and 30 (upper line and symbols) and ω_γ_ = 0

**Fig. 4:**
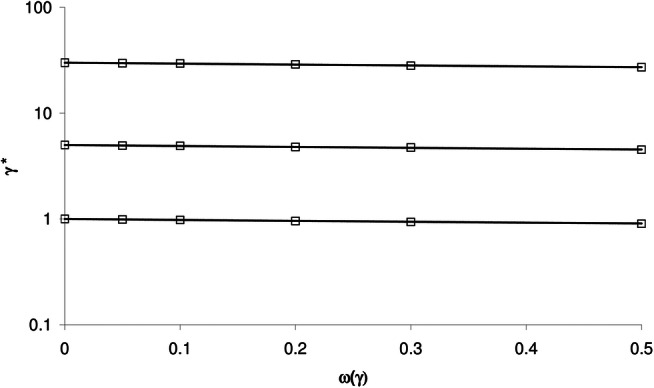
Relationship between γ* and ω_γ_ obtained by Monte Carlo simulation (open symbols), and the model prediction using Eq. (11) (solid lines), for γ = 1 (lower line and symbols), 5 and 30 (upper line and symbols) and ω_C50_ = 0

Figure 3 shows an example of the relationship between γ* and ω_C50_ obtained by Monte Carlo simulation (open symbols), and the model prediction using Eq. (11) (solid lines), for γ = 1, 5 and 30 and ω_γ_ = 0. In contrast to ω_C50_, the influence of ω_γ_ on γ* is rather small; as shown in Fig. 4, γ* is hardly affected by ω_γ_. Interestingly, the influence of ω_C50_ and ω_γ_ is rather small and comparable for γ = 1, but for γ = 30, the effect of ω_C50_ on γ* (and σ*) is much larger than that of ω_γ_. This differential effect is well predicted by Eqs. (10) and (11).

To confirm the validity of Eqs. (10) and (11), the simulated data sets were analyzed also using the cumulative log-normal distribution function (Eqs. (2) and (3)) instead of the sigmoid E_max_ model. As shown in Table I (complete results in supplemental table S1), the estimated values of σ were close to the values σ* calculated using Eq. (10), also with a mean precision of 1.2% (range − 4.7 to 4.5%).

Using Eqs. (11) and (10), the example in Figs. 1 and 2 (γ = 30, σ = 0.0567, ω_C50_ = 0.1, ω_γ_ = 0.1) results in γ* = 5.27 and σ* = 0.323, respectively.

### Procedure 2: Simulation and Population Analysis Using NONMEM

#### Estimation of IIV in C50 and γ

To investigate whether the parameters C50 and γ as well as their IIV (ω_C50_ and ω_γ_) could be estimated from clinical study data with acceptable precision, a series of simulations was performed using NONMEM. Each simulation consisted of 1000 runs with 40 individuals each, and 4 binary observations in each individual (see methods section for details). The results have been summarized in Table II (complete results in supplemental table S2).

**Table II:** Median (95% Confidence Interval) of 1000 Runs Estimating Parameters C50 and γ and Their IIV (ω_C50_ and ω_γ_). Binary Data (40 Individuals with 4 Observations in Each Individual) were Obtained from Simulations with Varying Population Values of γ, ω_C50_ and ω_γ_ (Complete Results in supplemental table S2)

Simulation			Estimation							
γ	ω_C50_	ω_γ_	C50	95% CI^a^	γ	95% CI^*a*^	ω_C50_	ω_γ_	#minim^*b*^	#covar^*c*^
1	0	0	1.00	0.66–1.61	1.06	0.78–1.72	0^*d*^	0^*d*^	958	140
1	0	0.1	1.00	0.66–1.57	1.05	0.75–2.40	0^*d*^	0.069	930	145
1	0.1	0	1.00	0.66–1.58	1.05	0.77–2.52	0.059	0^*d*^	962	163
1	0.1	0.1	1.00	0.64–1.53	1.03	0.75–2.80	0^*d*^	0.051	939	161
5	0	0	1.00	0.91–1.09	5.38	3.97–10.6	0^*d*^	0^*d*^	985	145
5	0	0.1	1.00	0.92–1.09	5.27	3.77–13.6	0^*d*^	0.069	970	136
5	0.1	0	1.00	0.88–1.14	5.41	3.68–47.0	0.092	0^*d*^	904	206
5	0.1	0.1	1.00	0.87–1.14	5.16	3.39–46.9	0.079	0.017	922	278
30	0	0	1.00	0.99–1.01	32.0	23.2–49.8^*e*^	0^*d*^	0^*d*^	957	118
30	0	0.1	1.00	0.98–1.01	32.0	22.9–49.8^*e*^	0^*d*^	0.063	925	131
30	0.1	0	1.00	0.99–1.01	49.8^*d*^	*e*	0.416	0^*d*^	747	0
30	0.1	0.1	1.00	0.99–1.01	49.8^*d*^	*e*	0.425	0^*d*^	772	0

^*a*^95% confidence interval

^*b*^Number of runs with successful minimization (out of 1000 runs for each simulation)

^*c*^Number of runs with successful covariance step (out of 1000 runs for each simulation)

^*d*^Value near lower boundary

^*e*^Value near upper boundary

In all simulations, median C50 was very close to the true value for each tested value of γ (1, 5, 30), but 95% confidence intervals of C50 were rather wide for low values of γ. Median γ was overestimated by about 6%. For γ = 30 and IIV in C50 during simulation, γ became very high, resulting in a “near boundary” message in NONMEM. Minimization was successful in most runs, but often the covariance step was not performed, in most cases due to a “near boundary” occurrence.

When IIV was absent during simulation, the estimate of the corresponding variance became very low, resulting in a “near boundary” message in NONMEM, indicating the absence of variance in the corresponding parameter. In most cases where IIV was present during simulation, the corresponding variance could be estimated, but the estimated values were far from the variance used in the simulations. Therefore it can be concluded that the tested study design (40 individuals with 4 binary observations in each individual) is not suited to estimate IIV in C50 and γ with reasonable precision.

#### Estimation of IIV in C50

A similar series of simulations were performed assuming that IIV in γ was absent during simulation and estimation. The results have been summarized in Table III (complete results in supplemental table S3). Comparing the results of Tables II and III, it can be concluded that the influence of IIV in γ is small compared to that of IIV in C50. However, for γ = 5 and ω_γ_ = 0.5, the estimated value for γ (18.8) is far from its true value (supplemental table S3). In addition, accuracy of estimates of ω_C50_ is poor. For example, for γ = 30 and ω_C50_ = 0.1 during simulation, the estimated ω_C50_ = 0.413 (Table III).

**Table III:** Median (95% Confidence Interval) of 1000 Runs Estimating Parameters C50 and γ and IIV in C50 (ω_C50_). IIV in γ Was Assumed to Be Absent During Estimation (ω_γ_ = 0). Binary Data (40 Individuals with 4 Observations in each Individual) Were Obtained from Simulations with Varying Population Values of γ, ω_C50_, and ω_γ_ (Complete Results in supplemental table S3)

Simulation			Estimation							
γ	ω_C50_	ω_γ_	C50	95% CI^*a*^	γ	95% CI^*a*^	ω_C50_	ω_γ_	#minim^*b*^	#covar^*c*^
1	0	0	1.00	0.67–1.57	1.05	0.78–1.47	0^*d*^	0^*e*^	1000	462
1	0	0.1	1.00	0.66–1.51	1.02	0.75–1.49	0^*d*^	0^*e*^	1000	380
1	0.1	0	1.00	0.66–1.58	1.04	0.77–1.50	0.098	0^*e*^	1000	563
1	0.1	0.1	1.00	0.66–1.51	1.01	0.75–1.47	0^*d*^	0^*e*^	998	475
5	0	0	1.00	0.91–1.09	5.32	3.98–7.56	0^*d*^	0^*e*^	999	476
5	0	0.1	1.00	0.92–1.09	5.16	3.77–7.34	0^*d*^	0^*e*^	1000	375
5	0.1	0	1.00	0.88–1.15	5.19	3.64–31.2	0.096	0^*e*^	977	970
5	0.1	0.1	1.00	0.87–1.15	4.79	3.31–30.9	0.089	0^*e*^	986	962
30	0	0	1.00	0.99–1.01	31.6	23.2–45.0	0^*d*^	0^*e*^	1000	425
30	0	0.1	1.00	0.99–1.01	30.8	23.1–46.0^*f*^	0^*d*^	0^*e*^	1000	406
30	0.1	0	1.00	0.99–1.01	49.8^*f*^		0.413	0^*e*^	947	17
30	0.1	0.1	1.00	0.99–1.01	49.8^*f*^	45.9–49.8^*f*^	0.418	0^*e*^	935	58

^*a*^95% confidence interval

^*b*^Number of runs with successful minimization (out of 1000 runs for each simulation)

^*c*^Number of runs with successful covariance step (out of 1000 runs for each simulation)

^*d*^Value near lower boundary

^*e*^Fixed value

^*f*^Value near upper boundary

#### Estimation without IIV

In Table IV the results of estimation without IIV (naive pooling approach) are shown (complete results in supplemental table S4). Minimization and covariance step were successful in almost all runs, irrespective of the values of γ, ω_C50_, and ω_γ_.

**Table IV:** Median (95% Confidence Interval) of 1000 Runs Estimating Parameters C50 and γ, Assuming Absence of IIV in C50 and γ (ω_C50_ = 0 and ω_γ_ = 0, Corresponding to a Naive Pooling Approach) During Estimation Step. Binary Data (40 Individuals with 4 Observations in Each Individual) Were Obtained from Simulations with Varying Population Values of γ, ω_C50_ and ω_γ_. γ* Is Calculated from Eq. (11) (Complete Results in supplemental table S4)

Simulation			Estimation							
γ	ω_C50_	ω_γ_	C50	95% CI^*a*^	γ	95% CI^*a*^	γ*	%diff^*b*^	#minim^*c*^	#covar^*d*^
1	0	0	1.00	0.67–1.57	1.01	0.77–1.34	1.00	− 1.0	1000	1000
1	0	0.1	1.00	0.66–1.52	1.00	0.74–1.32	0.98	− 2.1	1000	1000
1	0.1	0	1.00	0.66–1.58	0.99	0.74–1.29	0.98	− 0.4	1000	1000
1	0.1	0.1	1.00	0.66–1.53	0.98	0.74–1.30	0.96	− 2.1	1000	1000
5	0	0	1.00	0.91–1.09	5.11	3.79–6.69	5.00	− 2.2	1000	1000
5	0	0.1	1.00	0.92–1.10	5.00	3.71–6.56	4.90	− 2.3	1000	1000
5	0.1	0	1.00	0.87–1.16	3.67	2.79–4.69	3.66	− 0.2	1000	1000
5	0.1	0.1	1.00	0.87–1.15	3.58	2.71–4.80	3.58	0.0	1000	1000
30	0	0	1.00	0.99–1.02	30.6	23.1–40.1	30.0	− 2.0	998	998
30	0	0.1	1.00	0.99–1.01	30.0	22.3–39.7	29.4	− 2.1	1000	1000
30	0.1	0	1.00	0.89–1.12	5.32	3.98–7.15	5.29	− 0.4	1000	1000
30	0.1	0	1.00	0.89–1.12	5.31	3.97–7.31	5.27	− 0.8	1000	1000

^*a*^95% confidence interval

^*b*^% difference between γ* and median estimated γ

^*c*^Number of runs with successful minimization (out of 1000 runs for each simulation)

^*d*^Number of runs with successful covariance step (out of 1000 runs for each simulation)

In the absence of IIV in C50, the results are broadly comparable to that in Table II, but the bias in γ is smaller (about 2% *versus* 6% in Table II) and confidence intervals of γ are narrower. As expected from the results in Table I, median γ decreases with increasing ω_C50_ and, to a much lesser degree, ω_γ_, and is close to γ* calculated using Eq. (11), with a mean precision of 1.9% (root mean squared error; range − 6.2 to + 2.0%) for any of the 48 tested combinations of γ, ω_C50_, and ω_γ_.

#### Influence of Number of Individuals and Number of Observations per Individual

Another set of simulations was performed to investigate whether the number of individuals or the number of binary observations in each individual may affect the relationship described in Eqs. (10) and (11). For convenience, ω_C50_ was fixed to 0.1 and ω_γ_ = 0 during simulation and IIV was assumed to be absent during estimation (naive pooling).

The results have been summarized in supplemental table S5. The median estimates of γ (0.98 to 1.01 for γ = 1; 3.60 to 3.67 for γ = 5; 5.25 to 5.41 for γ = 30) were close to the values γ* calculated from Eq. (11), which were 0.983, 3.66 and 5.29 for γ = 1, 5, and 30, respectively (Table I), irrespective of the number of individuals and the number of binary observations in each individual.

#### Simulations with Continuous Data

Finally, a set of simulations was performed to confirm that Eqs. (10) and (11) are valid for continuous data as well. Data sets were generated similar to that in Tables II, III, and IV, without the dichotomy step. Instead, random data error with mean zero and standard deviation (SD) 0.1 (corresponding to 10% of the full scale from 0 to 1) was added to the simulated values. IIV was assumed to be absent during estimation (naive pooling).

The results have been summarized in supplemental table S6. The median estimates of γ were close to the values γ* calculated from Eq. (11) (Table I), with a mean precision of 2.5% (root mean squared error; range − 7.6 to + 2.3%) for any of the 48 tested combinations of γ, ω_C50_ and ω_γ_. This demonstrates that Eqs. (10) and (11) are valid for continuous data as well as for binary data.

## DISCUSSION

The first aim was to describe quantitatively the relationship between the steepness of the concentration–effect relationship and IIV in the model parameters. To this purpose, we derived an empirical equation using the principle that variances sum up. According to this principle, we postulated that the variance σ*^2^ may be approximated by Eq. (7), summing σ^2^ and the variances ω_C50_ and ω_γ_ and the interaction of ω_C50_ and ω_γ_. Using a series of Monte Carlo simulations (procedure 1), the constants p_i_ in Eq. (7) were estimated, resulting in Eqs. (10) and (11), describing the combined effect of γ and IIV in C50 and γ on the population estimates σ* and γ*, respectively.

Note that Eq. (11) was derived using Eqs. (6) to (8), which are based on the similarity of the sigmoid E_max_ model and the cumulative log-normal distribution. The difference between both distributions, depicted in Figs. 1 and 2, is less than 0.01 (1%) over the entire scale and likely not distinguishable when applied to clinical data. Both distributions can be interconverted using Eq. (6). Note that Eq. (6) is an approximation, since the distributions are not identical. The close resemblance of both functions implies that both functions can be chosen in population analysis, without a clear preference.

The second aim was to investigate whether IIV in the model parameters can be estimated by population modeling with data obtained from study designs as used in reported clinical research studies (9–12). From our simulations (procedure 2) it can be concluded that the tested study design (40 individuals with 4 binary observations in each individual) is not suited to estimate IIV in both C50 and γ with reasonable precision. Assuming that IIV in γ is absent during estimation results in slightly more precise estimates of C50, γ, and IIV in C50. Using a naive pooling procedure, *i.e.*, assuming absence of IIV in all parameters during estimation, results in more precise estimates. In the case that IIV is present during simulation, the estimated population estimates γ* are significantly lower than the value of γ used for simulation.

The effect of model parameters and their IIV on parameter estimates has been described in a few Monte Carlo simulation studies and with binary data (3, 13) and continuous data (14, 15). Lu and colleagues investigated the reliability of pharmacodynamic analysis by logistic regression. Some of their findings were confirmed in our study, *e.g.*, the good accuracy of the estimates of C50 and the minor impact of inclusion or exclusion of IIV in γ (13). They stated that, when data from multiple patients is naively pooled, the estimates of γ may be biased, and the 95% confidence intervals may not contain the true value. The authors stated in the legend to their figure 9: “A possible explanation of how the estimate of steepness of the concentration-effect relation (γ) may be biased when data from multiple patients is pooled for analysis. In this example, single data points are taken from each of nine different patients, each of whom have a steep concentration-effect relation but different values of drug concentration associated with a 50% probability of drug effect (C50) The resultant pooled concentration-effect curve appears flat (i.e., the apparent value of γ is lower than the true value.” and in the conclusion they stated: “When we simulated pooled data from multiple patients (with log-normal distributions for C50 and γ), there was a larger bias in γ estimates (up to 30%), even when n was large and %CI was significantly smaller” (3). The results of Lu and colleagues are supported by the findings in the present paper. However, we believe that the qualification “bias” should not be used here. Instead of stating that the estimate of γ is biased when obtained from a pooled analysis, we propose to state that the steepness is reflected in the population estimate obtained from a pooled analysis, denoted γ* in our paper. This parameter γ* is not the steepness of the concentration–effect relationship in an individual. Instead, it describes the population-predicted value of P as a function of the concentration, and therefore it predicts the probability of drug effect in an arbitrary individual, which can be used in pharmacokinetic-pharmacodynamic information displays that allow bedside prediction of the probability of response to standardized stimulus, such as the commercially available SmartPilot® View (Draeger, Germany) and Navigator Applications Suite (GE Healthcare, USA). In contrast, estimating γ as well as IIV in C50 and γ provides a value of γ that is not directly suited for predicting drug effect in an arbitrary individual, since it does not take into account the inter-individual variability in C50 and γ. For this reason we used a naive pooling approach in the development of a triple interaction model to estimate the potency of a combination of sevoflurane, propofol, and remifentanil (12).

Our simulations show that it does not seem easy to design and perform a study to estimate C50 and γ as well as their IIV. In the data of the investigated studies (9–11) we found several occasions where patients were responding to a stimulus after showing tolerance to that stimulus at a lower drug level, suggesting that γ is not very high; if γ is very high, such observation would be very unlikely. On the other hand, such observations may be a result of the preceding stimuli, and thus a methodological shortcoming of the study design. In addition, it remains to be determined whether γ is essentially identical for each patient, or that it includes significant IIV. Besides, it may be noted that the value of γ cannot be determined precisely in all cases, since its value becomes very high during the population analysis with IIV, as shown in clinical studies (9, 12) as well as in the simulations in the current paper. A full analysis of optimal study design is out of the scope of the present paper. More information on sample size calculations can be found in Ogungbenro and Aarons (16) and a practical example can be found in the supplemental digital content to Weerink *et al.* (17, 18).

In our simulations, we included 4 observations per individual. It is obvious that the location of the 4 observations per individual is important. It would have been possible to select an optimal design for each set of simulations. This would imply an extremely heavy burden on computer time and would require the implementation of an efficient procedure for optimal design. Instead, we choose a fixed design of 4 observations per individual, chosen in such a way that the “true” probability of drug effect P is 0.1, 0.25, 0.75, and 0.9 for each set of simulations.

In conclusion: An empirical equation (Eq. (11)) was derived describing the steepness of the population-predicted concentration–effect profile (γ*) as a function of γ and the IIV in C50 and γ and was validated for both binary and continuous data. The tested study design (40 individuals with 4 binary observations in each individual) is not suited to estimate the IIV in C50 and γ with reasonable precision. Using a naive pooling procedure, the population estimates γ* are significantly lower than the value of γ used for simulation. The steepness of the population-predicted concentration–effect relationship (γ*) is less than that of the individuals (γ). Using γ*, the population-predicted drug effect represents the drug effect, for binary data the probability of a drug effect, at a given concentration for an arbitrary individual. Therefore using γ* is better suited to clinical tools, *e.g.*, in anesthesia.

## Supplementary Information

ESM 1 (PDF 181 kb)
