# Supplementary material for: Population Pharmacodynamic Modeling Using the Sigmoid Emax Model: Influence of Inter-individual Variability on the Steepness of the Concentration–Effect Relationship. a Simulation Study
Source: AAPS J. 2020 Dec 24;23(1):10. doi: 10.1208/s12248-020-00549-7 (PMC7759489; doi:10.1208/s12248-020-00549-7)
Supplement: Supplementary file 1 — (PDF 181 kb) [file 12248_2020_549_MOESM1_ESM.pdf]

Supplemental data to

Population pharmacodynamic modeling: Influence of inter-individual variability on the steepness of the concentration – effect relationship. A simulation study

Abbreviations in tables S1-S6

#	set number
#indiv	number of individuals
#obs	number of observations per individual
simulation	
γ	typical value of γ
ωC50	variance of C50
$\omega\gamma$	variance of γ
estimation	
γ_{mc}	γ obtained by Monte Carlo simulation
γ^*	calculated from eq. (11)
σ_{mc}	σ obtained by Monte Carlo simulation
σ^*	calculated from eq. (10)
C50	median C50
γ	median γ
SD	median SD
CI_lower	lower value of 95% confidence interval
CI_upper	upper value of 95% confidence interval
#minim	number of sets with successful minimization step
#covar	number of sets with successful covariance step

Supplemental table S1. Comparison of γ and σ estimated by fitting to the sum of 10,000 simulated probability profiles of the sigmoid E_{max} model (γ_{mc}) and cumulative log-normal distribution (σ_{mc}) and calculated from eqs. (11) (γ*) and (10) (σ*), respectively. %diff is the % difference between γ* and γ_{mc} or between σ* and σ_{mc}.

	simulation			estimation					
#	γ	ωC50	$\omega\gamma$	γ_{mc}	γ*	%diff	σ_{mc}	σ*	%diff
1	0.5	0	0	0.500	0.500	0.0	3.396	3.400	0.1
2	0.5	0	0.05	0.496	0.495	-0.3	3.423	3.437	0.4
3	0.5	0	0.1	0.490	0.490	-0.1	3.466	3.473	0.2
4	0.5	0	0.2	0.481	0.480	-0.3	3.530	3.544	0.4
5	0.5	0	0.3	0.467	0.470	0.8	3.640	3.614	-0.7
6	0.5	0	0.5	0.448	0.453	1.2	3.795	3.750	-1.2
7	0.5	0.05	0	0.499	0.499	0.0	3.404	3.407	0.1
8	0.5	0.05	0.05	0.495	0.493	-0.2	3.433	3.446	0.4
9	0.5	0.05	0.1	0.489	0.488	-0.2	3.474	3.484	0.3
10	0.5	0.05	0.2	0.478	0.478	-0.1	3.549	3.558	0.3
11	0.5	0.05	0.3	0.464	0.468	1.0	3.664	3.631	-0.9
12	0.5	0.05	0.5	0.449	0.451	0.3	3.783	3.773	-0.3
13	0.5	0.1	0	0.498	0.498	0.0	3.412	3.415	0.1
14	0.5	0.1	0.05	0.492	0.492	-0.1	3.448	3.455	0.2
15	0.5	0.1	0.1	0.486	0.487	0.2	3.495	3.494	0.0
16	0.5	0.1	0.2	0.477	0.476	-0.3	3.558	3.572	0.4
17	0.5	0.1	0.3	0.468	0.466	-0.5	3.627	3.648	0.6
18	0.5	0.1	0.5	0.447	0.448	0.2	3.800	3.796	-0.1
19	0.5	0.2	0	0.495	0.496	0.1	3.429	3.429	0.0
20	0.5	0.2	0.05	0.490	0.490	0.0	3.468	3.473	0.1
21	0.5	0.2	0.1	0.485	0.484	-0.3	3.499	3.516	0.5
22	0.5	0.2	0.2	0.475	0.472	-0.6	3.575	3.600	0.7
23	0.5	0.2	0.3	0.466	0.462	-1.0	3.640	3.682	1.2
24	0.5	0.2	0.5	0.446	0.442	-0.7	3.811	3.842	0.8
25	0.5	0.3	0	0.493	0.494	0.2	3.447	3.444	-0.1
26	0.5	0.3	0.05	0.488	0.487	-0.2	3.480	3.491	0.3
27	0.5	0.3	0.1	0.482	0.481	-0.3	3.522	3.537	0.4
28	0.5	0.3	0.2	0.470	0.469	-0.3	3.611	3.628	0.5
29	0.5	0.3	0.3	0.459	0.457	-0.3	3.699	3.716	0.5
30	0.5	0.3	0.5	0.445	0.437	-1.8	3.814	3.887	1.9
31	0.5	0.5	0	0.488	0.490	0.2	3.477	3.473	-0.1
32	0.5	0.5	0.05	0.482	0.482	0.0	3.523	3.526	0.1
33	0.5	0.5	0.1	0.476	0.475	-0.3	3.565	3.579	0.4
34	0.5	0.5	0.2	0.467	0.462	-1.2	3.634	3.682	1.3
35	0.5	0.5	0.3	0.454	0.449	-1.1	3.737	3.783	1.2
36	0.5	0.5	0.5	0.442	0.428	-3.3	3.840	3.976	3.6
37	1	0	0	1.000	1.000	0.0	1.698	1.700	0.1
38	1	0	0.05	0.990	0.989	0.0	1.715	1.718	0.2
39	1	0	0.1	0.977	0.979	0.2	1.737	1.736	-0.1
40	1	0	0.2	0.956	0.959	0.3	1.776	1.772	-0.2
41	1	0	0.3	0.936	0.941	0.6	1.815	1.807	-0.5
42	1	0	0.5	0.902	0.907	0.5	1.883	1.875	-0.5

43	1	0.05	0	0.990	0.991	0.1	1.714	1.715	0.0
44	1	0.05	0.05	0.979	0.980	0.1	1.734	1.735	0.1
45	1	0.05	0.1	0.969	0.969	0.0	1.752	1.754	0.1
46	1	0.05	0.2	0.944	0.948	0.4	1.798	1.793	-0.3
47	1	0.05	0.3	0.933	0.928	-0.5	1.820	1.831	0.6
48	1	0.05	0.5	0.885	0.893	0.9	1.920	1.905	-0.8
49	1	0.1	0	0.981	0.983	0.2	1.730	1.729	-0.1
50	1	0.1	0.05	0.969	0.971	0.2	1.751	1.751	0.0
51	1	0.1	0.1	0.953	0.959	0.7	1.782	1.772	-0.6
52	1	0.1	0.2	0.931	0.937	0.7	1.824	1.814	-0.5
53	1	0.1	0.3	0.917	0.917	-0.1	1.851	1.855	0.2
54	1	0.1	0.5	0.889	0.879	-1.2	1.909	1.934	1.3
55	1	0.2	0	0.963	0.967	0.4	1.763	1.758	-0.3
56	1	0.2	0.05	0.952	0.954	0.2	1.783	1.783	0.0
57	1	0.2	0.1	0.941	0.941	0.0	1.804	1.807	0.2
58	1	0.2	0.2	0.924	0.917	-0.8	1.838	1.855	0.9
59	1	0.2	0.3	0.903	0.894	-1.0	1.880	1.901	1.1
60	1	0.2	0.5	0.863	0.854	-1.1	1.967	1.991	1.2
61	1	0.3	0	0.948	0.952	0.4	1.791	1.786	-0.3
62	1	0.3	0.05	0.931	0.937	0.7	1.824	1.814	-0.6
63	1	0.3	0.1	0.921	0.923	0.2	1.842	1.841	-0.1
64	1	0.3	0.2	0.904	0.897	-0.7	1.879	1.895	0.9
65	1	0.3	0.3	0.879	0.873	-0.7	1.931	1.947	0.8
66	1	0.3	0.5	0.841	0.831	-1.2	2.018	2.047	1.4
67	1	0.5	0	0.915	0.923	0.9	1.856	1.841	-0.8
68	1	0.5	0.05	0.904	0.907	0.3	1.877	1.875	-0.1
69	1	0.5	0.1	0.894	0.891	-0.3	1.899	1.908	0.5
70	1	0.5	0.2	0.873	0.862	-1.2	1.945	1.972	1.4
71	1	0.5	0.3	0.854	0.836	-2.1	1.988	2.035	2.4
72	1	0.5	0.5	0.817	0.789	-3.3	2.077	2.154	3.7
73	2	0	0	2.000	2.000	0.0	0.849	0.850	0.1
74	2	0	0.05	1.975	1.979	0.2	0.860	0.859	-0.1
75	2	0	0.1	1.966	1.958	-0.4	0.864	0.868	0.5
76	2	0	0.2	1.924	1.919	-0.3	0.883	0.886	0.4
77	2	0	0.3	1.900	1.882	-1.0	0.894	0.903	1.1
78	2	0	0.5	1.826	1.813	-0.7	0.930	0.937	0.8
79	2	0.05	0	1.928	1.934	0.3	0.880	0.879	-0.2
80	2	0.05	0.05	1.907	1.911	0.2	0.890	0.890	-0.1
81	2	0.05	0.1	1.886	1.889	0.2	0.900	0.900	0.0
82	2	0.05	0.2	1.839	1.847	0.4	0.923	0.921	-0.3
83	2	0.05	0.3	1.792	1.807	0.8	0.947	0.941	-0.7
84	2	0.05	0.5	1.730	1.735	0.3	0.981	0.980	-0.2
85	2	0.1	0	1.861	1.874	0.7	0.912	0.907	-0.6
86	2	0.1	0.05	1.847	1.850	0.1	0.919	0.919	0.0
87	2	0.1	0.1	1.815	1.827	0.6	0.935	0.931	-0.5
88	2	0.1	0.2	1.770	1.782	0.7	0.959	0.954	-0.5
89	2	0.1	0.3	1.742	1.741	0.0	0.975	0.977	0.2
90	2	0.1	0.5	1.659	1.666	0.4	1.023	1.020	-0.2
91	2	0.2	0	1.751	1.770	1.1	0.969	0.960	-0.9
92	2	0.2	0.05	1.722	1.744	1.3	0.986	0.975	-1.1
93	2	0.2	0.1	1.704	1.718	0.8	0.996	0.989	-0.7
94	2	0.2	0.2	1.663	1.671	0.5	1.021	1.017	-0.3
95	2	0.2	0.3	1.626	1.627	0.1	1.043	1.045	0.1

96	2	0.2	0.5	1.559	1.549	-0.6	1.088	1.097	0.8
97	2	0.3	0	1.660	1.681	1.3	1.023	1.011	-1.1
98	2	0.3	0.05	1.633	1.654	1.2	1.039	1.028	-1.1
99	2	0.3	0.1	1.610	1.627	1.1	1.054	1.045	-0.9
100	2	0.3	0.2	1.566	1.578	0.8	1.083	1.077	-0.6
101	2	0.3	0.3	1.529	1.534	0.3	1.110	1.108	-0.1
102	2	0.3	0.5	1.457	1.454	-0.2	1.164	1.169	0.4
103	2	0.5	0	1.511	1.538	1.7	1.123	1.106	-1.6
104	2	0.5	0.05	1.489	1.509	1.3	1.140	1.127	-1.2
105	2	0.5	0.1	1.471	1.482	0.7	1.154	1.147	-0.6
106	2	0.5	0.2	1.424	1.432	0.5	1.191	1.187	-0.3
107	2	0.5	0.3	1.404	1.386	-1.3	1.208	1.226	1.5
108	2	0.5	0.5	1.338	1.307	-2.3	1.268	1.300	2.6
109	3	0	0	3.000	3.000	0.0	0.566	0.567	0.1
110	3	0	0.05	2.957	2.968	0.4	0.574	0.573	-0.2
111	3	0	0.1	2.928	2.937	0.3	0.580	0.579	-0.2
112	3	0	0.2	2.876	2.878	0.1	0.590	0.591	0.0
113	3	0	0.3	2.783	2.822	1.4	0.610	0.602	-1.3
114	3	0	0.5	2.736	2.720	-0.6	0.621	0.625	0.6
115	3	0.05	0	2.766	2.791	0.9	0.614	0.609	-0.7
116	3	0.05	0.05	2.726	2.757	1.1	0.623	0.617	-1.0
117	3	0.05	0.1	2.697	2.725	1.1	0.629	0.624	-0.9
118	3	0.05	0.2	2.636	2.664	1.1	0.644	0.638	-0.9
119	3	0.05	0.3	2.571	2.607	1.4	0.660	0.652	-1.2
120	3	0.05	0.5	2.454	2.503	2.0	0.692	0.679	-1.8
121	3	0.1	0	2.588	2.620	1.2	0.656	0.649	-1.1
122	3	0.1	0.05	2.552	2.586	1.3	0.665	0.657	-1.2
123	3	0.1	0.1	2.526	2.553	1.1	0.672	0.666	-0.9
124	3	0.1	0.2	2.468	2.492	0.9	0.688	0.682	-0.8
125	3	0.1	0.3	2.408	2.434	1.1	0.705	0.698	-0.9
126	3	0.1	0.5	2.300	2.330	1.3	0.737	0.730	-1.1
127	3	0.2	0	2.320	2.355	1.5	0.732	0.722	-1.4
128	3	0.2	0.05	2.286	2.321	1.5	0.743	0.732	-1.4
129	3	0.2	0.1	2.254	2.289	1.5	0.753	0.743	-1.3
130	3	0.2	0.2	2.197	2.228	1.4	0.773	0.763	-1.2
131	3	0.2	0.3	2.161	2.172	0.5	0.785	0.783	-0.3
132	3	0.2	0.5	2.061	2.071	0.5	0.823	0.821	-0.2
133	3	0.3	0	2.129	2.157	1.3	0.797	0.788	-1.2
134	3	0.3	0.05	2.098	2.124	1.3	0.809	0.800	-1.1
135	3	0.3	0.1	2.073	2.093	1.0	0.819	0.812	-0.8
136	3	0.3	0.2	2.011	2.034	1.1	0.844	0.836	-0.9
137	3	0.3	0.3	1.966	1.979	0.7	0.863	0.859	-0.5
138	3	0.3	0.5	1.889	1.882	-0.4	0.898	0.903	0.6
139	3	0.5	0	1.849	1.876	1.4	0.918	0.906	-1.3
140	3	0.5	0.05	1.830	1.845	0.8	0.928	0.921	-0.7
141	3	0.5	0.1	1.792	1.816	1.3	0.947	0.936	-1.1
142	3	0.5	0.2	1.761	1.761	0.0	0.964	0.966	0.2
143	3	0.5	0.3	1.719	1.710	-0.5	0.988	0.994	0.6
144	3	0.5	0.5	1.665	1.622	-2.6	1.019	1.048	2.9
145	5	0	0	5.000	5.000	0.0	0.340	0.340	0.1
146	5	0	0.05	4.935	4.947	0.2	0.344	0.344	-0.1
147	5	0	0.1	4.906	4.895	-0.2	0.346	0.347	0.3
148	5	0	0.2	4.794	4.797	0.1	0.354	0.354	0.0

149	5	0	0.3	4.734	4.704	-0.6	0.359	0.361	0.7
150	5	0	0.5	4.516	4.534	0.4	0.376	0.375	-0.3
151	5	0.05	0	4.116	4.178	1.5	0.412	0.407	-1.3
152	5	0.05	0.05	4.077	4.131	1.3	0.416	0.412	-1.2
153	5	0.05	0.1	3.995	4.086	2.3	0.425	0.416	-2.1
154	5	0.05	0.2	3.903	4.000	2.5	0.435	0.425	-2.3
155	5	0.05	0.3	3.854	3.920	1.7	0.440	0.434	-1.5
156	5	0.05	0.5	3.662	3.772	3.0	0.463	0.451	-2.7
157	5	0.1	0	3.610	3.661	1.4	0.470	0.464	-1.3
158	5	0.1	0.05	3.583	3.619	1.0	0.474	0.470	-0.9
159	5	0.1	0.1	3.492	3.579	2.5	0.486	0.475	-2.3
160	5	0.1	0.2	3.406	3.502	2.8	0.498	0.485	-2.6
161	5	0.1	0.3	3.323	3.430	3.2	0.511	0.496	-2.9
162	5	0.1	0.5	3.237	3.299	1.9	0.524	0.515	-1.7
163	5	0.2	0	2.965	3.026	2.1	0.573	0.562	-1.9
164	5	0.2	0.05	2.940	2.991	1.7	0.577	0.568	-1.6
165	5	0.2	0.1	2.924	2.957	1.1	0.581	0.575	-1.0
166	5	0.2	0.2	2.856	2.892	1.2	0.594	0.588	-1.1
167	5	0.2	0.3	2.792	2.831	1.4	0.608	0.600	-1.2
168	5	0.2	0.5	2.689	2.720	1.2	0.631	0.625	-0.9
169	5	0.3	0	2.602	2.637	1.3	0.652	0.645	-1.2
170	5	0.3	0.05	2.577	2.606	1.1	0.659	0.652	-1.0
171	5	0.3	0.1	2.530	2.576	1.8	0.671	0.660	-1.6
172	5	0.3	0.2	2.495	2.519	1.0	0.680	0.675	-0.8
173	5	0.3	0.3	2.456	2.465	0.4	0.691	0.690	-0.2
174	5	0.3	0.5	2.366	2.368	0.1	0.717	0.718	0.1
175	5	0.5	0	2.138	2.167	1.3	0.794	0.785	-1.2
176	5	0.5	0.05	2.112	2.141	1.4	0.804	0.794	-1.2
177	5	0.5	0.1	2.087	2.116	1.4	0.814	0.803	-1.2
178	5	0.5	0.2	2.071	2.068	-0.1	0.820	0.822	0.2
179	5	0.5	0.3	2.042	2.024	-0.9	0.832	0.840	1.0
180	5	0.5	0.5	1.977	1.943	-1.7	0.859	0.875	1.9
181	10	0	0	10.000	10.000	0.0	0.170	0.170	0.1
182	10	0	0.05	9.898	9.894	0.0	0.172	0.172	0.2
183	10	0	0.1	9.769	9.791	0.2	0.174	0.174	-0.1
184	10	0	0.2	9.548	9.594	0.5	0.178	0.177	-0.4
185	10	0	0.3	9.400	9.408	0.1	0.181	0.181	0.0
186	10	0	0.5	9.171	9.067	-1.1	0.185	0.187	1.2
187	10	0.05	0	5.943	6.052	1.8	0.286	0.281	-1.7
188	10	0.05	0.05	5.849	6.005	2.7	0.290	0.283	-2.4
189	10	0.05	0.1	5.790	5.958	2.9	0.293	0.285	-2.7
190	10	0.05	0.2	5.671	5.869	3.5	0.299	0.290	-3.2
191	10	0.05	0.3	5.516	5.784	4.9	0.308	0.294	-4.4
192	10	0.05	0.5	5.347	5.623	5.2	0.317	0.302	-4.7
193	10	0.1	0	4.663	4.735	1.6	0.364	0.359	-1.4
194	10	0.1	0.05	4.643	4.701	1.2	0.366	0.362	-1.1
195	10	0.1	0.1	4.621	4.668	1.0	0.367	0.364	-0.9
196	10	0.1	0.2	4.497	4.603	2.4	0.378	0.369	-2.2
197	10	0.1	0.3	4.431	4.541	2.5	0.383	0.374	-2.3
198	10	0.1	0.5	4.277	4.424	3.4	0.397	0.384	-3.1
199	10	0.2	0	3.537	3.553	0.5	0.480	0.478	-0.3
200	10	0.2	0.05	3.496	3.529	1.0	0.486	0.482	-0.8
201	10	0.2	0.1	3.477	3.506	0.8	0.488	0.485	-0.7

202	10	0.2	0.2	3.472	3.460	-0.4	0.489	0.491	0.5
203	10	0.2	0.3	3.382	3.416	1.0	0.502	0.498	-0.9
204	10	0.2	0.5	3.320	3.333	0.4	0.511	0.510	-0.2
205	10	0.3	0	2.937	2.964	0.9	0.578	0.573	-0.8
206	10	0.3	0.05	2.966	2.945	-0.7	0.573	0.577	0.8
207	10	0.3	0.1	2.891	2.926	1.2	0.587	0.581	-1.0
208	10	0.3	0.2	2.900	2.888	-0.4	0.586	0.589	0.5
209	10	0.3	0.3	2.842	2.853	0.4	0.597	0.596	-0.2
210	10	0.3	0.5	2.816	2.785	-1.1	0.603	0.610	1.2
211	10	0.5	0	2.305	2.338	1.4	0.736	0.727	-1.3
212	10	0.5	0.05	2.336	2.323	-0.6	0.727	0.732	0.7
213	10	0.5	0.1	2.271	2.308	1.6	0.747	0.737	-1.4
214	10	0.5	0.2	2.277	2.279	0.1	0.746	0.746	0.0
215	10	0.5	0.3	2.308	2.251	-2.5	0.736	0.755	2.6
216	10	0.5	0.5	2.248	2.199	-2.2	0.756	0.773	2.3
217	20	0	0	20.000	20.000	0.0	0.085	0.085	0.1
218	20	0	0.05	19.728	19.787	0.3	0.086	0.086	-0.2
219	20	0	0.1	19.581	19.581	0.0	0.087	0.087	0.1
220	20	0	0.2	19.313	19.187	-0.7	0.088	0.089	0.8
221	20	0	0.3	18.627	18.816	1.0	0.091	0.090	-0.9
222	20	0	0.5	18.186	18.135	-0.3	0.093	0.094	0.3
223	20	0.05	0	7.060	7.107	0.7	0.240	0.239	-0.5
224	20	0.05	0.05	7.026	7.078	0.7	0.242	0.240	-0.6
225	20	0.05	0.1	6.899	7.049	2.2	0.246	0.241	-2.0
226	20	0.05	0.2	6.938	6.993	0.8	0.245	0.243	-0.7
227	20	0.05	0.3	6.792	6.938	2.1	0.250	0.245	-2.0
228	20	0.05	0.5	6.594	6.832	3.6	0.257	0.249	-3.3
229	20	0.1	0	5.149	5.192	0.8	0.330	0.327	-0.7
230	20	0.1	0.05	5.168	5.173	0.1	0.329	0.329	0.0
231	20	0.1	0.1	5.113	5.154	0.8	0.332	0.330	-0.7
232	20	0.1	0.2	5.077	5.118	0.8	0.334	0.332	-0.7
233	20	0.1	0.3	5.016	5.082	1.3	0.338	0.335	-1.2
234	20	0.1	0.5	5.000	5.012	0.3	0.340	0.339	-0.1
235	20	0.2	0	3.708	3.734	0.7	0.458	0.455	-0.6
236	20	0.2	0.05	3.706	3.722	0.4	0.458	0.457	-0.3
237	20	0.2	0.1	3.689	3.709	0.6	0.460	0.458	-0.5
238	20	0.2	0.2	3.701	3.685	-0.4	0.459	0.461	0.5
239	20	0.2	0.3	3.680	3.661	-0.5	0.461	0.464	0.7
240	20	0.2	0.5	3.599	3.614	0.4	0.472	0.470	-0.3
241	20	0.3	0	3.043	3.067	0.8	0.558	0.554	-0.7
242	20	0.3	0.05	3.065	3.057	-0.3	0.554	0.556	0.3
243	20	0.3	0.1	3.109	3.047	-2.0	0.546	0.558	2.1
244	20	0.3	0.2	3.011	3.027	0.6	0.564	0.562	-0.4
245	20	0.3	0.3	3.046	3.008	-1.3	0.558	0.565	1.4
246	20	0.3	0.5	3.039	2.970	-2.3	0.559	0.572	2.4
247	20	0.5	0	2.372	2.387	0.6	0.716	0.712	-0.5
248	20	0.5	0.05	2.385	2.379	-0.3	0.712	0.714	0.4
249	20	0.5	0.1	2.376	2.372	-0.2	0.715	0.717	0.3
250	20	0.5	0.2	2.377	2.357	-0.9	0.715	0.721	0.9
251	20	0.5	0.3	2.380	2.342	-1.6	0.714	0.726	1.7
252	20	0.5	0.5	2.358	2.313	-1.9	0.721	0.735	2.0
253	30	0	0	30.000	30.000	0.0	0.057	0.057	0.1
254	30	0	0.05	29.590	29.681	0.3	0.057	0.057	-0.2

255	30	0	0.1	29.524	29.372	-0.5	0.058	0.058	0.6
256	30	0	0.2	28.887	28.781	-0.4	0.059	0.059	0.5
257	30	0	0.3	28.088	28.225	0.5	0.060	0.060	-0.4
258	30	0	0.5	27.132	27.202	0.3	0.063	0.062	-0.2
259	30	0.05	0	7.418	7.370	-0.7	0.229	0.231	0.8
260	30	0.05	0.05	7.312	7.351	0.5	0.232	0.231	-0.4
261	30	0.05	0.1	7.300	7.331	0.4	0.233	0.232	-0.3
262	30	0.05	0.2	7.304	7.294	-0.1	0.232	0.233	0.3
263	30	0.05	0.3	7.124	7.257	1.9	0.238	0.234	-1.7
264	30	0.05	0.5	6.984	7.185	2.9	0.243	0.237	-2.7
265	30	0.1	0	5.290	5.292	0.0	0.321	0.321	0.1
266	30	0.1	0.05	5.267	5.279	0.2	0.322	0.322	-0.1
267	30	0.1	0.1	5.244	5.267	0.4	0.324	0.323	-0.3
268	30	0.1	0.2	5.159	5.242	1.6	0.329	0.324	-1.5
269	30	0.1	0.3	5.201	5.218	0.3	0.327	0.326	-0.2
270	30	0.1	0.5	5.120	5.171	1.0	0.332	0.329	-0.9
271	30	0.2	0	3.743	3.771	0.7	0.454	0.451	-0.6
272	30	0.2	0.05	3.783	3.763	-0.5	0.449	0.452	0.6
273	30	0.2	0.1	3.757	3.755	-0.1	0.452	0.453	0.2
274	30	0.2	0.2	3.748	3.738	-0.3	0.453	0.455	0.4
275	30	0.2	0.3	3.757	3.722	-0.9	0.452	0.457	1.0
276	30	0.2	0.5	3.723	3.690	-0.9	0.456	0.461	1.0
277	30	0.3	0	3.068	3.087	0.6	0.553	0.551	-0.5
278	30	0.3	0.05	3.079	3.081	0.1	0.551	0.552	0.1
279	30	0.3	0.1	3.056	3.074	0.6	0.556	0.553	-0.5
280	30	0.3	0.2	3.087	3.061	-0.9	0.550	0.555	0.9
281	30	0.3	0.3	3.042	3.048	0.2	0.559	0.558	-0.1
282	30	0.3	0.5	3.040	3.022	-0.6	0.559	0.562	0.7
283	30	0.5	0	2.419	2.396	-0.9	0.702	0.709	1.0
284	30	0.5	0.05	2.366	2.391	1.1	0.718	0.711	-0.9
285	30	0.5	0.1	2.409	2.386	-1.0	0.705	0.712	1.1
286	30	0.5	0.2	2.380	2.376	-0.2	0.713	0.715	0.3
287	30	0.5	0.3	2.359	2.366	0.3	0.720	0.718	-0.2
288	30	0.5	0.5	2.337	2.347	0.4	0.726	0.724	-0.3
289	50	0	0	50.000	50.000	0.0	0.034	0.034	0.1
290	50	0	0.05	49.471	49.468	0.0	0.034	0.034	0.1
291	50	0	0.1	49.028	48.953	-0.2	0.035	0.035	0.2
292	50	0	0.2	49.189	47.968	-2.5	0.035	0.035	2.6
293	50	0	0.3	48.273	47.041	-2.6	0.035	0.036	2.6
294	50	0	0.5	47.423	45.337	-4.4	0.036	0.037	4.5
295	50	0.05	0	7.598	7.516	-1.1	0.224	0.226	1.2
296	50	0.05	0.05	7.463	7.505	0.6	0.228	0.227	-0.5
297	50	0.05	0.1	7.511	7.494	-0.2	0.226	0.227	0.3
298	50	0.05	0.2	7.541	7.473	-0.9	0.225	0.227	1.0
299	50	0.05	0.3	7.401	7.451	0.7	0.229	0.228	-0.6
300	50	0.05	0.5	7.441	7.408	-0.4	0.228	0.229	0.5
301	50	0.1	0	5.333	5.345	0.2	0.318	0.318	-0.1
302	50	0.1	0.05	5.274	5.338	1.2	0.322	0.318	-1.1
303	50	0.1	0.1	5.242	5.331	1.7	0.324	0.319	-1.5
304	50	0.1	0.2	5.251	5.316	1.2	0.323	0.320	-1.1
305	50	0.1	0.3	5.280	5.302	0.4	0.322	0.321	-0.3
306	50	0.1	0.5	5.308	5.274	-0.7	0.320	0.322	0.8
307	50	0.2	0	3.748	3.790	1.1	0.453	0.449	-1.0

308	50	0.2	0.05	3.706	3.785	2.2	0.458	0.449	-1.9
309	50	0.2	0.1	3.789	3.781	-0.2	0.448	0.450	0.3
310	50	0.2	0.2	3.750	3.771	0.6	0.453	0.451	-0.4
311	50	0.2	0.3	3.847	3.761	-2.2	0.441	0.452	2.4
312	50	0.2	0.5	3.715	3.742	0.7	0.457	0.454	-0.6
313	50	0.3	0	3.096	3.098	0.0	0.549	0.549	0.0
314	50	0.3	0.05	3.098	3.094	-0.1	0.548	0.549	0.3
315	50	0.3	0.1	3.035	3.090	1.8	0.559	0.550	-1.6
316	50	0.3	0.2	3.063	3.082	0.6	0.554	0.552	-0.5
317	50	0.3	0.3	3.107	3.074	-1.1	0.547	0.553	1.2
318	50	0.3	0.5	3.073	3.059	-0.5	0.553	0.556	0.6
319	50	0.5	0	2.412	2.401	-0.5	0.704	0.708	0.6
320	50	0.5	0.05	2.388	2.398	0.4	0.711	0.709	-0.4
321	50	0.5	0.1	2.380	2.395	0.7	0.714	0.710	-0.6
322	50	0.5	0.2	2.370	2.389	0.8	0.717	0.712	-0.7
323	50	0.5	0.3	2.389	2.383	-0.2	0.711	0.713	0.3
324	50	0.5	0.5	2.376	2.371	-0.2	0.714	0.717	0.3

Supplemental table S2. Median (95% confidence interval) of 1000 runs estimating parameters C50 and γ and their IIV (ω_{C50} and ω_{γ}). Binary data (40 individuals with 4 observations in each individual) were obtained from simulations with varying population values of γ, ω_{C50} and ω_{γ}.

	simulation			estimation									
#	γ	ωC50	ωγ	C50	CI_lower	CI_upper	γ	CI_lower	CI_upper	ωC50	ωγ	#minim	#covar
1	1	0	0	1.00	0.66	1.61	1.06	0.78	1.72	0.000	0.000	958	140
2	1	0	0.02	1.00	0.64	1.56	1.05	0.78	1.94	0.000	0.000	957	130
3	1	0	0.05	1.00	0.63	1.50	1.06	0.77	2.83	0.000	0.031	953	141
4	1	0	0.1	1.00	0.66	1.57	1.05	0.75	2.40	0.000	0.069	930	145
5	1	0	0.2	1.00	0.62	1.53	1.03	0.72	2.81	0.000	0.112	909	136
6	1	0	0.5	1.01	0.59	3.05	1.02	0.69	2.74	0.000	0.228	884	83
7	1	0.02	0	1.00	0.64	1.58	1.07	0.80	2.36	0.000	0.000	966	147
8	1	0.05	0	1.00	0.66	1.50	1.07	0.78	2.39	0.000	0.000	959	147
9	1	0.1	0	1.00	0.66	1.58	1.05	0.77	2.52	0.059	0.000	962	163
10	1	0.2	0	1.00	0.65	1.60	1.06	0.75	2.81	0.140	0.000	967	179
11	1	0.5	0	1.00	0.61	1.61	1.05	0.76	2.96	0.509	0.000	963	231
12	1	0.02	0.02	1.00	0.65	1.55	1.06	0.77	2.20	0.000	0.000	955	128
13	1	0.05	0.05	1.00	0.65	1.58	1.06	0.76	2.62	0.000	0.008	963	163
14	1	0.1	0.1	1.00	0.64	1.53	1.03	0.75	2.80	0.000	0.051	939	161
15	1	0.2	0.2	1.00	0.62	1.60	1.02	0.71	3.27	0.000	0.087	931	169
16	1	0.5	0.5	1.00	0.59	1.83	0.93	0.64	3.53	0.000	0.176	898	161
17	5	0	0	1.00	0.91	1.09	5.38	3.97	10.61	0.000	0.000	985	145
18	5	0	0.02	1.00	0.92	1.09	5.31	3.86	8.06	0.000	0.000	979	151
19	5	0	0.05	1.00	0.91	1.09	5.22	3.89	12.44	0.000	0.028	965	153
20	5	0	0.1	1.00	0.92	1.09	5.27	3.77	13.61	0.000	0.069	970	136
21	5	0	0.2	1.00	0.90	1.09	5.24	3.72	15.52	0.000	0.114	952	134
22	5	0	0.5	1.00	0.91	1.10	5.03	3.47	14.72	0.000	0.212	931	106
23	5	0.02	0	1.00	0.91	1.11	5.15	3.77	14.28	0.018	0.000	974	196
24	5	0.05	0	1.00	0.89	1.13	5.19	3.61	15.70	0.043	0.000	946	220

25	5	0.1	0	1.00	0.88	1.14	5.41	3.68	47.00	0.092	0.000	904	206
26	5	0.2	0	1.00	0.83	1.15	5.38	3.56	40.10	0.229	0.000	810	263
27	5	0.5	0	1.00	0.87	1.14	26.80	3.35	28.62	5.490	2.410	729	391
28	5	0.02	0.02	1.00	0.91	1.11	5.14	3.70	16.30	0.017	0.000	973	210
29	5	0.05	0.05	1.00	0.89	1.12	5.12	3.57	49.50	0.044	0.000	945	232
30	5	0.1	0.1	1.00	0.87	1.14	5.16	3.39	46.90	0.079	0.017	922	278
31	5	0.2	0.2	1.00	0.84	1.18	5.65	3.06	39.93	0.196	0.213	868	307
32	5	0.5	0.5	1.00	0.86	1.24	23.70	2.65	27.30	6.295	2.860	844	434
33	30	0	0	1.00	0.99	1.01	32.05	23.20	49.80	0.000	0.000	957	118
34	30	0	0.02	1.00	0.98	1.02	31.80	23.10	49.80	0.000	0.010	951	135
35	30	0	0.05	1.00	0.99	1.01	32.10	22.50	49.80	0.000	0.023	949	131
36	30	0	0.1	1.00	0.99	1.01	32.00	22.89	49.80	0.000	0.063	925	131
37	30	0	0.2	1.00	0.99	1.02	31.40	22.02	49.80	0.000	0.104	889	127
38	30	0	0.5	1.00	0.98	1.02	31.75	20.70	49.80	0.000	0.230	837	72
39	30	0.02	0	1.00	0.98	1.02	49.80	20.44	49.80	0.028	0.067	959	3
40	30	0.05	0	1.00	0.99	1.01	49.80	49.80	49.80	0.110	0.000	941	0
41	30	0.1	0	1.00	0.99	1.01	49.80	49.80	49.80	0.416	0.000	747	0
42	30	0.2	0	1.00	0.96	1.04	46.00	44.80	48.00	1.710	0.000	586	4
43	30	0.5	0	1.00	0.93	1.07	29.20	28.40	30.70	4.200	0.000	444	4
44	30	0.02	0.02	1.00	0.98	1.02	49.80	19.80	49.80	0.028	0.033	962	1
45	30	0.05	0.05	1.00	0.99	1.01	49.80	49.80	49.80	0.112	0.000	945	0
46	30	0.1	0.1	1.00	0.99	1.01	49.80	49.80	49.80	0.425	0.000	772	0
47	30	0.2	0.2	1.00	0.96	1.05	45.70	44.50	47.80	1.730	0.000	597	23
48	30	0.5	0.5	1.00	0.94	1.07	28.70	27.80	30.00	4.300	0.000	466	28

Supplemental table S3. Median (95% confidence interval) of 1000 runs estimating parameters C50 and γ and IIV in C50 (ω_{C50}). IIV in γ was assumed to be absent during estimation ($\omega_{\gamma} = 0$). Binary data (40 individuals with 4 observations in each individual) were obtained from simulations with varying population values of γ, ω_{C50} and ω_{γ}.

	simulation			estimation									
#	γ	ωC50	ωγ	C50	CI_lower	CI_upper	γ	CI_lower	CI_upper	ωC50	ωγ	#minim	#covar
1	1	0	0	1.00	0.67	1.57	1.05	0.79	1.47	0.000	0	1000	462
2	1	0	0.02	1.00	0.66	1.50	1.04	0.78	1.49	0.000	0	999	461
3	1	0	0.05	1.00	0.64	1.46	1.04	0.78	1.56	0.000	0	1000	441
4	1	0	0.1	1.00	0.67	1.51	1.02	0.75	1.49	0.000	0	1000	380
5	1	0	0.2	1.00	0.65	1.51	1.00	0.72	1.51	0.000	0	999	373
6	1	0	0.5	1.00	0.64	1.57	0.95	0.68	1.43	0.000	0	999	260
7	1	0.02	0	1.00	0.66	1.57	1.06	0.80	1.53	0.004	0	1000	502
8	1	0.05	0	1.00	0.67	1.49	1.05	0.78	1.50	0.010	0	999	505
9	1	0.1	0	1.00	0.66	1.58	1.04	0.77	1.50	0.098	0	1000	563
10	1	0.2	0	1.00	0.66	1.59	1.04	0.76	1.56	0.149	0	996	609
11	1	0.5	0	1.00	0.62	1.58	1.03	0.76	1.53	0.459	0	995	775
12	1	0.02	0.02	1.00	0.66	1.51	1.05	0.78	1.50	0.000	0	999	454
13	1	0.05	0.05	1.00	0.66	1.55	1.04	0.76	1.49	0.000	0	998	481
14	1	0.1	0.1	1.00	0.66	1.51	1.01	0.75	1.47	0.000	0	998	475
15	1	0.2	0.2	1.00	0.64	1.56	0.98	0.71	1.52	0.000	0	999	474
16	1	0.5	0.5	1.00	0.61	1.68	0.88	0.63	1.43	0.000	0	998	478
17	5	0	0	1.00	0.91	1.09	5.33	3.98	7.56	0.000	0	999	476
18	5	0	0.02	1.00	0.92	1.09	5.21	3.86	7.38	0.000	0	1000	447
19	5	0	0.05	1.00	0.92	1.09	5.13	3.89	7.43	0.000	0	1000	443
20	5	0	0.1	1.00	0.92	1.09	5.16	3.77	7.34	0.000	0	1000	375
21	5	0	0.2	1.00	0.92	1.08	5.06	3.70	7.29	0.000	0	1000	370
22	5	0	0.5	1.00	0.92	1.08	4.75	3.49	6.73	0.000	0	1000	279
23	5	0.02	0	1.00	0.91	1.10	5.11	3.77	7.71	0.018	0	1000	781
24	5	0.05	0	1.00	0.90	1.13	5.06	3.59	8.56	0.045	0	999	927

25	5	0.1	0	1.00	0.88	1.15	5.19	3.64	31.21	0.096	0	977	970
26	5	0.2	0	1.00	0.83	1.15	5.19	3.50	36.20	0.221	0	952	935
27	5	0.5	0	1.00	0.86	1.18	18.85	3.35	27.10	1.800	0	949	899
28	5	0.02	0.02	1.00	0.91	1.10	5.09	3.70	7.55	0.016	0	999	758
29	5	0.05	0.05	1.00	0.89	1.13	4.97	3.57	8.59	0.045	0	998	927
30	5	0.1	0.1	1.00	0.87	1.15	4.79	3.31	30.90	0.089	0	986	962
31	5	0.2	0.2	1.00	0.84	1.19	4.39	2.98	26.09	0.188	0	972	962
32	5	0.5	0.5	1.00	0.80	1.27	3.58	2.23	24.10	0.568	0	969	949
33	30	0	0	1.00	0.99	1.01	31.60	23.20	45.01	0.000	0	1000	425
34	30	0	0.02	1.00	0.99	1.02	31.20	23.10	43.60	0.000	0	1000	433
35	30	0	0.05	1.00	0.99	1.01	31.45	22.50	44.21	0.000	0	998	428
36	30	0	0.1	1.00	0.99	1.01	30.80	23.09	46.01	0.000	0	1000	406
37	30	0	0.2	1.00	0.99	1.01	30.20	22.10	43.50	0.000	0	1000	346
38	30	0	0.5	1.00	0.99	1.01	28.50	20.10	40.71	0.000	0	999	257
39	30	0.02	0	1.00	0.97	1.03	49.80	20.00	49.80	0.028	0	992	56
40	30	0.05	0	1.00	0.99	1.01	49.80	49.80	49.80	0.110	0	980	1
41	30	0.1	0	1.00	0.99	1.01	49.80	49.80	49.80	0.413	0	947	17
42	30	0.2	0	1.00	0.96	1.04	46.00	44.80	48.00	1.710	0	888	679
43	30	0.5	0	1.00	0.93	1.07	29.20	28.40	30.70	4.200	0	923	640
44	30	0.02	0.02	1.00	0.98	1.02	49.80	19.79	49.80	0.028	0	994	69
45	30	0.05	0.05	1.00	0.99	1.01	49.80	49.80	49.80	0.112	0	986	5
46	30	0.1	0.1	1.00	0.99	1.01	49.80	45.90	49.80	0.418	0	935	58
47	30	0.2	0.2	1.00	0.96	1.05	45.60	33.00	47.50	1.730	0	918	669
48	30	0.5	0.5	1.00	0.94	1.07	28.60	20.70	29.81	4.250	0	905	637

Supplemental table S4. Median (95% confidence interval) of 1000 runs estimating parameters C50 and γ, assuming absence of IIV in C50 and γ ($\omega_{C50} = 0$ and $\omega_{\gamma} = 0$, corresponding to a naive pooling approach) during estimation step. Binary data (40 individuals with 4 observations in each individual) were obtained from simulations with varying population values of γ, ω_{C50} and ω_{γ}. γ^* is calculated from eq. (11) and %diff is the % difference between γ^* and median estimated γ.

	simulation			estimation									
	γ	ωC50	$\omega\gamma$	C50	CI_lower	CI_upper	γ	CI_lower	CI_upper	γ^*	%diff	#minim	#covar
1	1	0	0	1.00	0.67	1.57	1.01	0.77	1.34	1.00	-1.0	1000	1000
2	1	0	0.02	1.00	0.66	1.51	1.00	0.77	1.31	1.00	-0.3	1000	1000
3	1	0	0.05	1.00	0.64	1.46	1.01	0.76	1.33	0.99	-2.1	1000	1000
4	1	0	0.1	1.00	0.66	1.52	1.00	0.74	1.32	0.98	-2.1	1000	1000
5	1	0	0.2	1.00	0.65	1.52	0.97	0.72	1.34	0.96	-1.5	1000	1000
6	1	0	0.5	1.00	0.64	1.57	0.93	0.68	1.28	0.91	-2.5	1000	1000
7	1	0.02	0	1.00	0.65	1.57	1.01	0.78	1.35	1.00	-1.3	1000	1000
8	1	0.05	0	1.00	0.67	1.50	1.01	0.76	1.35	0.99	-1.4	1000	1000
9	1	0.1	0	1.00	0.66	1.58	0.99	0.74	1.29	0.98	-0.4	1000	1000
10	1	0.2	0	1.00	0.65	1.59	0.99	0.73	1.31	0.97	-2.0	1000	1000
11	1	0.5	0	1.00	0.61	1.62	0.93	0.70	1.22	0.92	-1.2	1000	1000
12	1	0.02	0.02	1.00	0.66	1.51	1.00	0.76	1.33	0.99	-0.8	1000	1000
13	1	0.05	0.05	1.00	0.66	1.58	0.99	0.74	1.31	0.98	-1.0	1000	1000
14	1	0.1	0.1	1.00	0.66	1.53	0.98	0.74	1.30	0.96	-2.1	1000	1000
15	1	0.2	0.2	1.00	0.64	1.56	0.96	0.69	1.26	0.92	-4.2	1000	1000
16	1	0.5	0.5	1.00	0.60	1.68	0.84	0.62	1.16	0.79	-6.4	1000	1000
17	5	0	0	1.00	0.91	1.09	5.11	3.79	6.69	5.00	-2.2	1000	1000
18	5	0	0.02	1.00	0.92	1.09	5.01	3.75	6.53	4.98	-0.6	1000	1000
19	5	0	0.05	1.00	0.91	1.09	4.95	3.80	6.62	4.95	-0.1	1000	1000
20	5	0	0.1	1.00	0.92	1.10	5.01	3.71	6.59	4.90	-2.3	1000	1000
21	5	0	0.2	1.00	0.92	1.08	4.93	3.63	6.59	4.80	-2.7	1000	1000
22	5	0	0.5	1.00	0.92	1.08	4.65	3.42	6.39	4.53	-2.4	1000	1000
23	5	0.02	0	1.00	0.91	1.11	4.62	3.49	6.03	4.62	-0.1	1000	1000

24	5	0.05	0	1.00	0.89	1.13	4.18	3.15	5.51	4.18	-0.1	1000	1000
25	5	0.1	0	1.00	0.87	1.16	3.67	2.79	4.69	3.66	-0.2	1000	1000
26	5	0.2	0	1.00	0.82	1.19	3.03	2.28	3.95	3.03	-0.1	1000	1000
27	5	0.5	0	1.00	0.76	1.29	2.17	1.60	2.90	2.17	-0.2	1000	1000
28	5	0.02	0.02	1.00	0.91	1.11	4.65	3.48	6.02	4.60	-1.1	1000	1000
29	5	0.05	0.05	1.00	0.89	1.13	4.13	3.13	5.29	4.13	0.0	1000	1000
30	5	0.1	0.1	1.00	0.87	1.15	3.58	2.71	4.80	3.58	0.0	1000	1000
31	5	0.2	0.2	1.00	0.83	1.20	2.90	2.23	3.88	2.89	-0.3	1000	1000
32	5	0.5	0.5	1.00	0.77	1.35	2.04	1.49	2.65	1.94	-4.9	1000	1000
33	30	0	0	1.00	0.99	1.02	30.60	23.09	40.10	30.00	-2.0	998	998
34	30	0	0.02	1.00	0.99	1.02	30.50	23.00	40.00	29.87	-2.1	1000	1000
35	30	0	0.05	1.00	0.99	1.01	30.30	22.40	40.40	29.68	-2.1	1000	1000
36	30	0	0.1	1.00	0.99	1.01	30.00	22.30	39.70	29.37	-2.1	1000	1000
37	30	0	0.2	1.00	0.99	1.01	29.50	21.70	40.80	28.78	-2.5	1000	999
38	30	0	0.5	1.00	0.99	1.01	28.10	20.10	37.60	27.20	-3.2	1000	1000
39	30	0.02	0	1.00	0.95	1.05	11.20	8.49	15.10	11.16	-0.4	1000	1000
40	30	0.05	0	1.00	0.92	1.08	7.46	5.45	10.20	7.37	-1.2	1000	998
41	30	0.1	0	1.00	0.89	1.12	5.32	3.98	7.15	5.29	-0.4	1000	1000
42	30	0.2	0	1.00	0.85	1.17	3.73	0.80	5.27	3.77	1.2	1000	938
43	30	0.5	0	1.00	0.76	1.31	2.35	0.80	3.29	2.40	2.0	987	840
44	30	0.02	0.02	1.00	0.95	1.05	11.20	8.16	14.70	11.14	-0.5	1000	1000
45	30	0.05	0.05	1.00	0.93	1.08	7.36	5.52	9.90	7.35	-0.1	1000	999
46	30	0.1	0.1	1.00	0.89	1.12	5.31	3.94	7.31	5.27	-0.8	1000	1000
47	30	0.2	0.2	1.00	0.85	1.20	3.76	0.80	5.01	3.74	-0.6	1000	946
48	30	0.5	0.5	1.00	0.69	1.29	2.30	0.80	3.26	2.35	2.0	969	738

Supplemental table S5. Median (95% confidence interval) of 1000 runs estimating parameters C50 and γ. Binary data were obtained from simulations with varying population values of γ and varying number of individuals (#indiv) and number of observations per individual (#obs). During simulation ω_{C50} was fixed to 0.1 and ω_{γ} = 0. IIV was assumed to be absent during estimation (naive pooling). The % difference (%diff) between γ* calculated from eq. (11) and median estimated γ was small. γ* is 0.983, 3.66 and 5.29 for γ = 1, 5, and 30, respectively, irrespective of the number of individuals and the number of binary observations in each individual.

simulation			estimation									
#indiv	#obs	γ	C50	CI_lower	CI_upper	γ	CI_lower	CI_upper	γ*	%diff	#minim	#covar
40	4	1	1.00	0.65	1.52	1.01	0.76	1.32	0.98	-2.2	1000	1000
100	4	1	1.00	0.75	1.30	0.98	0.84	1.17	0.98	0.0	1000	1000
200	4	1	1.01	0.83	1.22	0.99	0.88	1.11	0.98	-0.4	1000	1000
400	4	1	1.00	0.87	1.14	0.98	0.90	1.07	0.98	0.0	1000	1000
40	4	5	1.00	0.87	1.16	3.67	2.79	4.69	3.66	-0.2	1000	1000
100	4	5	1.00	0.91	1.09	3.62	3.04	4.29	3.66	1.1	1000	1000
200	4	5	1.00	0.94	1.07	3.62	3.19	4.07	3.66	1.3	1000	1000
400	4	5	1.00	0.96	1.04	3.60	3.32	3.91	3.66	1.7	1000	1000
40	4	30	1.00	0.90	1.12	5.31	3.96	7.27	5.29	-0.3	1000	1000
100	4	30	1.00	0.94	1.07	5.29	4.39	6.46	5.29	0.0	1000	1000
200	4	30	1.00	0.95	1.05	5.25	4.62	5.99	5.29	0.8	1000	999
400	4	30	1.00	0.97	1.03	5.25	4.80	5.74	5.29	0.8	1000	1000
40	4	1	1.00	0.65	1.52	1.01	0.76	1.32	0.98	-2.2	1000	1000
40	10	1	1.00	0.77	1.27	0.99	0.80	1.20	0.98	-0.2	1000	1000
40	20	1	1.00	0.83	1.24	0.99	0.86	1.14	0.98	-0.6	1000	1000
40	40	1	1.00	0.85	1.16	0.98	0.88	1.09	0.98	0.1	1000	1000
40	4	5	1.00	0.87	1.16	3.67	2.79	4.69	3.66	-0.2	1000	1000
40	10	5	1.00	0.89	1.12	3.63	3.01	4.46	3.66	0.9	1000	1000

40	20	5	1.00	0.90	1.11	3.66	3.07	4.30	3.66	0.0	1000	1000
40	40	5	1.00	0.90	1.10	3.64	3.14	4.20	3.66	0.6	1000	1000
40	4	30	1.00	0.90	1.12	5.31	3.96	7.27	5.29	-0.3	1000	1000
40	10	30	1.00	0.90	1.10	5.41	4.22	7.02	5.29	-2.1	1000	998
40	20	30	1.00	0.90	1.11	5.41	4.23	7.05	5.29	-2.2	1000	998
40	40	30	1.00	0.90	1.10	5.36	4.14	7.05	5.29	-1.3	1000	999

Supplemental table S6. Median (95% confidence interval) of 1000 runs estimating parameters C50, γ and residual standard deviation (SD) from simulated continuous data. Data sets were generated similar to that in table 4, except for the dichotomy step. Instead, random data error with mean zero and standard deviation 0.1 (corresponding to 10% of the full scale from 0 to 1) was added to the simulated values. IIV was assumed to be absent during estimation (naive pooling). The % difference (%diff) between γ^* calculated from eq. (11) and median estimated γ was small, demonstrating that eqs. (10) and (11) are valid for both binary and continuous data.

	simulation			estimation										
#	γ	ωC50	$\omega\gamma$	C50	CI_lower	CI_upper	γ	CI_lower	CI_upper	SD	γ^*	%diff	#minim	#covar
1	1	0	0	1.00	0.90	1.11	1.00	0.93	1.08	0.099	1.00	0.0	1000	1000
2	1	0	0.02	1.00	0.90	1.12	1.00	0.91	1.09	0.103	1.00	-0.2	1000	1000
3	1	0	0.05	1.00	0.91	1.11	1.00	0.90	1.10	0.108	0.99	-0.8	1000	1000
4	1	0	0.1	1.00	0.89	1.11	0.99	0.89	1.14	0.116	0.98	-1.4	1000	1000
5	1	0	0.2	1.00	0.91	1.12	0.98	0.84	1.15	0.128	0.96	-2.2	1000	1000
6	1	0	0.5	1.00	0.89	1.13	0.95	0.77	1.16	0.150	0.91	-4.5	1000	1000
7	1	0.02	0	1.00	0.88	1.11	1.00	0.93	1.07	0.101	1.00	0.1	1000	1000
8	1	0.05	0	1.00	0.87	1.14	0.99	0.91	1.07	0.104	0.99	-0.1	1000	1000
9	1	0.1	0	1.00	0.86	1.15	0.98	0.91	1.06	0.110	0.98	0.5	1000	1000
10	1	0.2	0	1.00	0.84	1.19	0.96	0.89	1.04	0.117	0.97	0.6	1000	1000
11	1	0.5	0	1.00	0.78	1.27	0.92	0.84	0.99	0.139	0.92	0.9	1000	1000
12	1	0.02	0.02	1.00	0.89	1.12	0.99	0.90	1.08	0.105	0.99	0.0	1000	1000
13	1	0.05	0.05	1.00	0.88	1.12	0.99	0.89	1.10	0.113	0.98	-0.6	1000	1000
14	1	0.1	0.1	1.01	0.87	1.15	0.98	0.86	1.09	0.123	0.96	-1.7	1000	1000
15	1	0.2	0.2	0.99	0.84	1.19	0.94	0.82	1.09	0.137	0.92	-2.7	1000	1000
16	1	0.5	0.5	1.00	0.79	1.27	0.84	0.70	1.02	0.163	0.79	-6.3	1000	1000
17	5	0	0	1.00	0.98	1.02	5.00	4.62	5.43	0.099	5.00	0.0	1000	1000
18	5	0	0.02	1.00	0.98	1.02	4.99	4.57	5.41	0.103	4.98	-0.2	1000	1000
19	5	0	0.05	1.00	0.98	1.02	4.98	4.50	5.51	0.109	4.95	-0.7	1000	1000
20	5	0	0.1	1.00	0.98	1.02	4.97	4.38	5.66	0.115	4.90	-1.5	1000	1000
21	5	0	0.2	1.00	0.98	1.02	4.92	4.22	5.73	0.127	4.80	-2.5	1000	1000

22	5	0	0.5	1.00	0.98	1.02	4.71	3.87	5.88	0.150	4.53	-3.7	1000	1000
23	5	0.02	0	1.00	0.95	1.05	4.59	4.20	4.99	0.139	4.62	0.6	1000	1000
24	5	0.05	0	1.00	0.92	1.08	4.10	3.69	4.53	0.177	4.18	1.9	1000	1000
25	5	0.1	0	1.00	0.90	1.10	3.58	3.11	4.05	0.213	3.66	2.3	1000	1000
26	5	0.2	0	1.00	0.87	1.16	2.98	2.49	3.50	0.250	3.03	1.5	1000	1000
27	5	0.5	0	1.00	0.79	1.26	2.16	1.70	2.65	0.293	2.17	0.3	1000	1000
28	5	0.02	0.02	1.00	0.95	1.05	4.54	4.16	4.97	0.141	4.60	1.2	1000	1000
29	5	0.05	0.05	1.00	0.92	1.08	4.06	3.61	4.62	0.177	4.13	1.7	1000	1000
30	5	0.1	0.1	1.00	0.91	1.12	3.51	3.03	4.10	0.212	3.58	2.0	1000	1000
31	5	0.2	0.2	1.00	0.85	1.16	2.86	2.39	3.41	0.246	2.89	1.1	1000	1000
32	5	0.5	0.5	1.01	0.78	1.26	1.99	1.60	2.45	0.277	1.94	-2.4	1000	1000
33	30	0	0	1.00	1.00	1.00	29.90	27.60	32.40	0.099	30.00	0.3	1000	1000
34	30	0	0.02	1.00	1.00	1.00	29.90	27.40	32.90	0.103	29.87	-0.1	1000	1000
35	30	0	0.05	1.00	1.00	1.00	29.90	27.10	33.20	0.108	29.68	-0.7	1000	1000
36	30	0	0.1	1.00	1.00	1.00	29.80	26.40	33.90	0.116	29.37	-1.4	1000	1000
37	30	0	0.2	1.00	1.00	1.00	29.40	25.70	34.10	0.127	28.78	-2.1	1000	1000
38	30	0	0.5	1.00	1.00	1.00	28.40	23.40	34.90	0.149	27.20	-4.2	1000	1000
39	30	0.02	0	1.00	0.92	1.09	11.70	8.71	49.80	0.310	11.16	-4.6	1000	821
40	30	0.05	0	1.01	0.87	1.14	7.98	5.67	49.80	0.342	7.37	-7.6	1000	719
41	30	0.1	0	1.00	0.88	1.13	5.31	4.00	7.58	0.350	5.29	-0.3	1000	977
42	30	0.2	0	1.00	0.76	1.31	3.81	2.64	47.01	0.362	3.77	-1.0	999	907
43	30	0.5	0	1.00	0.65	1.53	2.42	1.77	32.70	0.368	2.40	-1.0	999	942
44	30	0.02	0.02	1.00	0.92	1.09	11.60	8.41	49.80	0.310	11.14	-3.9	1000	827
45	30	0.05	0.05	1.00	0.87	1.14	7.80	5.78	49.80	0.341	7.35	-5.8	1000	747
46	30	0.1	0.1	1.00	0.88	1.13	5.30	4.00	9.47	0.348	5.27	-0.6	1000	972
47	30	0.2	0.2	0.99	0.76	1.31	3.84	2.83	45.00	0.357	3.74	-2.5	1000	908
48	30	0.5	0.5	1.00	0.74	1.50	2.42	1.77	31.41	0.363	2.35	-3.0	1000	961
